# Assessing the Identity of Commercial Herbs From a Cambodian Market Using DNA Barcoding

**DOI:** 10.3389/fphar.2020.00244

**Published:** 2020-03-24

**Authors:** Xinyun Cui, Weijie Li, Jianhe Wei, Yaodong Qi, Rongtao Li, Yun Yang, Yuhua Shi, Xiangxiao Meng, Yaolei Mi, Theang Huot, Wei Sun, Xilong Zheng

**Affiliations:** ^1^Hainan Provincial Key Laboratory of Resources Conservation and Development of Southern Medicine, Hainan Branch of the Institute of Medicinal Plant Development, Chinese Academy of Medical Sciences and Peking Union Medical College, Haikou, China; ^2^Institute of Medicinal Plant Development, Chinese Academy of Medical Sciences and Peking Union Medical College, Beijing, China; ^3^Key Laboratory of Beijing for Identification and Safety Evaluation of Chinese Medicine, Institute of Chinese Materia Medica, China Academy of Chinese Medical Sciences, Beijing, China; ^4^National Center of Traditional Medicine, Ministry of Health of Cambodia, Phnom Penh, Cambodia

**Keywords:** Cambodia, herb, DNA barcoding, ITS2, *psb*A-*trn*H

## Abstract

In Cambodia, medicinal plants are often used to treat various illnesses. However, the identities of many medicinal plants remain unknown. In this study, we collected 50 types of traditional Cambodian medicinal plants that could not be identified by their appearance from a domestic market. We utilized the DNA barcoding technique, combined with the literature survey, to trace their identities. In the end, 33 species were identified at the species level and 7 species were identified at the genus level. The ethnopharmacological information of 33 medicinal plants was documented. The DNA barcoding technique is useful in the identification of medicinal plants with no previous information.

## Introduction

Cambodia is located in the Indo-China Peninsula, where it borders Thailand, Vietnam, and Lao PDR in Southeast Asia. Although it does not exceed 4% (181,035 km^2^) of the total area of Southeast Asia, Cambodia is well-known for its rich biodiversity, overlapping with four of the 25 “biodiversity hotspots” and maintaining rich natural resources and a unique ecosystem. It is estimated that the country has more than 3,000 vascular plant species (Chassagne et al., 2016). Approximately 1,200 medicinal plants are used to treat diseases (Xu, 2008; Walker, 2017). Traditional medicine plays an important role in the lives of most Cambodians. In the face of disease, 70–80% of Cambodians opt for traditional medicinal methods (Walker, 2017; Yao et al., 2017) with approximately 40–50% of the population using medicinal plants daily (Chassagne et al., 2017).

Traditional Cambodian medicine involves several cultural and regional traditions derived from Theravada Buddhism, Ayurveda, traditional Chinese medicine, and French pharmaceutical traditions (Chassagne et al., 2017). Among these, Chinese and Ayurvedic medicines are the two oldest and most comprehensive medical systems based on natural medicinal agents. Consequently, the importance of traditional medicinal plant research in Cambodia is relatively high. However, Cambodia still does not have a national ethnopharmacopoeia (World Health Organization [WHO], 2005). Furthermore, there are few curricula teaching traditional Cambodian medicine, and books offer inconsistent and confusing information (Richman et al., 2010). Due to climate change and agro-industrial development in Cambodia, the local ecological environment is threatened (Chassagne et al., 2016). At the same time, Western medicine is being promoted, and knowledge of medicinal plants is being lost (Xu, 2008). Therefore, the study of medicinal plants in this country is very important. The medicinal plant market is not only a place for the sale of natural therapies but also a place for people to exchange information on medicinal plants, which preserves the knowledge as the information is passed from one generation to the next (Lee et al., 2008; de Carvalho Nilo Bitu et al., 2015; Jin et al., 2018). Therefore, we chose to conduct our study at a traditional market in Phnom Penh, the Cambodian capital.

**FIGURE 1:**
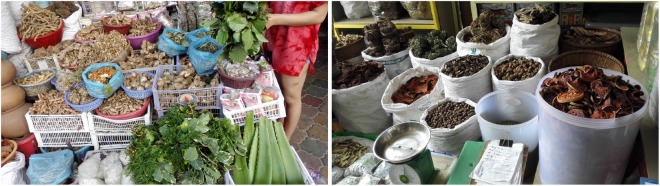
Stalls in Orussey Market selling medicinal plants.

We performed this study in August 2016, December 2016, and November 2017 in Orussey Market, which is one of the largest traditional markets for Chinese merchants in Phnom Penh carrying a wide variety of medicinal plants. The medicinal materials market that we surveyed represented only a small part of the entire Orussey Market (Figure 1). Only about 10 merchants were selling herbal medicines. The business model of the Cambodian medicinal plant market is mainly retail sales. Each store was small and independently run, with its own shop name. The medicinal plants were stored outside of the shops for customers to browse and purchase. The herbs had no fixed specifications, and they were derived from plants in the region. There were various types of herbs that included roots, stems, leaves, fruits, and whole plants. Most medicinal plants were previously dried, and a few fresh medicinal plants were formulated into medicines or single-flavored products. Due to the large number of Chinese customers, the shops also sold commonly used herbs in China, such as red dates, pepper, and *Atractylodes macrocephalae* rhizome (Atractylodes macrocephala Koidz.). The quality and specifications of the medicinal plants were not significantly different from store to store, and the price was similar across different shops. We collected samples from a total of 118 medicinal plants, of which 68 could be identified by morphology, whereas the remaining 50 species could not be morphologically distinguished. The main objective of this study was to clarify the original species of these medicinal plants.

To identify medicinal plants, morphological, microscopical, and physical and chemical identification methods are commonly used, but they require experienced investigators that are knowledgeable in the field. A shortage of experienced investigators has led to difficulties in identifying unknown plants. With advances in science and technology, such as chromatography, spectroscopy, and X-ray diffraction, new methods have been used to study medicinal plants (Chen et al., 2012). Although most methods are not useful when combined with the starting material of unknown origin, they can provide indirect evidence for the authenticity of the material (Han et al., 2016).

The DNA barcoding technique is an effective tool that can identify unknown medicinal plants with no background information (Chen S. et al., 2013). It uses a short DNA sequence from a standard and agreed-upon position in the genome to identify the species rapidly and accurately (de Vere et al., 2015; Li et al., 2015). The experimental method is fast, standardized, and simple. It generates a large experimental throughput and easily identifies the species (Chen et al., 2011). The common DNA barcodes for plants are *rbc*L, *mat*K, ITS2, and *psb*A-*trn*H; however, *mat*K is difficult to amplify with commonly used primers (Li et al., 2015); therefore, different taxonomic groups require different sets of primers (Hollingsworth, 2008). Furthermore, *mat*K sequences evolve slowly, and this locus has by far the lowest divergence among plastid genes in flowering plants (Kress et al., 2005). Due to its modest discriminatory ability, it is not recommended for studies at the species level. Presently, *psb*A-*trn*H is the most widely used plastid barcode for species identification, as its universal primers can amplify nearly all angiosperms (Shaw et al., 2007). Internal transcribed spacer 2 (ITS2), a part of the nuclear DNA, is another ideal barcode because of its short length, easy amplification with a single primer pair, high sequencing efficiency, and high variation between species (Yu et al., 2017).

Internal transcribed spacer 2 and *psb*A*-trn*H represent the universal barcode for the reliable identification of medicinal plants (Chen et al., 2010; Chen S. et al., 2013; Yao et al., 2010). The ITS2 sequence can accurately identify *Solani nigri* (Solanum nigrum L.) and its sibling species (Chen et al., 2017) *Scutellaria barbata* D.Don and its adulterants (Guo et al., 2016), and *Lycium barbarum* L. and its adulterants (Xin et al., 2013), as well as the origin of various ginseng plants based on SNP barcodes (Chen X. et al., 2013). This technique can also be used to classify and identify medicinal plants from family Orchidaceae (Tang et al., 2017). Furthermore, the ITS2 region has been used to supervise the proportions and varieties of adulterant species (Yu et al., 2017). Presently, DNA barcoding is widely used, as it has been applied in the authentication and identification of small berry fruits (Wu et al., 2018), as well as in the study of Li minority medicine (Cui et al., 2019) and various animal species (Yang et al., 2018). The technique has also been used to identify *Sida* L. herbal products (Santhosh Kumar et al., 2015), traditional Dai medicines, and laxative producing plants (Seethapathy et al., 2015). Han et al. (2016) identified unknown herbal plants in common markets and reported the adulterant rate. In this study, we used the DNA barcoding technique to identify the 50 unknown medicinal plants obtained from the Orussey Market, one of the largest Cambodian traditional markets.

## Results and Discussion

In total, we collected samples from 118 medicinal plants, of which 68 could be morphologically identified, the remaining 50 were randomly selected for DNA barcoding analysis. According to Table 1, 42 of the 50 samples were derived from the stem, bark, and vine, except for two fruits, four roots, one leaf, and one rhizome. Based on their morphological appearance, it was difficult to confirm their identity. The ITS2 amplification success rate was 94% (47/50), and the sequencing success rate was 98% (46/47). According to the results of ITS2 and *psb*A-*trn*H experiments, 33 plants were identified at the species level. Seven were identified at the genus level. Ten plants were unidentifiable (five were due to either amplification failures, sequencing failures or no matched results, and five were due to the low maximum similarity). There were 27 medicinal plants with a maximum similarity <97%, and the *psb*A-*trn*H region was amplified in these samples. The *psb*A-*trn*H amplification success rate was 52% (14/27), and the sequencing success rate was 100% (14/14). All the identification results (matched species, maximum similarity, length, and maximum score) are shown in Table 1. The maximum similarity range of ITS2 was 83–100%, and that for *psb*A-*trn*H was 89–100%. In this study, the ITS2 amplification of three samples and *psb*A-*trn*H amplification of 13 samples were unsuccessful, which might have been due to impure or degraded DNA of processed medicinal materials. This shows the boundedness of DNA barcoding due to the instability of DNA. This result also reflects the limitations of Sanger sequencing, such as the unspecific amplification of non-target DNA when target DNA is degraded (Pawar et al., 2017). In the method part, we have taken some measures to avoid these problems. If mixed sequencing signals were present, we would clone the PCR products and sequence the single colonies to identify target amplicons. To overcome the limitations of Sanger sequencing, we can employ next-generation sequencing (NGS) approach technology to simultaneously detect plant and fungal DNAs (Pawar et al., 2017). And it is also possible that the samples were not suitable for ITS2 or *psb*A-*trn*H amplification. To tackle this issue, specific primers (Zhao et al., 2018) or other types of barcodes, such as mini-barcodes (Song et al., 2017; Liu, 2018) or plastid super-barcodes (Krawczyk et al., 2018), can be used. However, this method also has limitations, according to our data, seven medicinal species were identified at the genus level, five exhibited low maximum similarity (<90%), and several could not be matched to any of the existing medicinal species, presumably due to the low species-level resolution of many plant genera and insufficient database information of GenBank. Therefore, the analyses with multiple genetic loci (e.g., single-nucleotide polymorphisms, SNPs) and other analytical methods, such as infrared spectroscopy and X-ray diffraction, must be employed to achieve high resolution for species differentiation (Chen et al., 2012).

**TABLE 1:** Herb identification results of ITS2 supplemented with *psb*A*-trn*H.

No.	Voucher number	Part used	ITS2 identification result	Maximum similarity (%)	Length (bp)	Max score	*psb*A*-trn*H identification result	Maximum similarity (%)	Length (bp)	Max score
JPZ01	20161214001	Stem bark	*Nauclea officinalis* (Pierre ex Pit.) Merr. & Chun	99	220	399	\^1^	\	\	\
JPZ02	20161214002	Stem	*Ficus sagittata* Vahl	100	202	374	\	\	\	\
JPZ03	20161214003	Stem	*Dalbergia pinnata* (Lour.) Prain	98	218	350	\	\	\	\
JPZ04	20161214004	Root	*Flacourtia indica* (Burm. f.) Merr.	96	220	357	*F. indica*	99	271	483
JPZ05	20161214005	Stem	*Anacardium occidentale* Linn.	99	229	418	\	\	\	\
JPZ06	20161214006	Root	*Calamus acanthospathus* Griff.	97	250	414	\	\	\	\
JPZ07	20161214007	Stem	Amplification failed	\	\	\	Amplification failed	\	\	\
JPZ08	20161214008	Stem	No significant similarity found	\	225	\	*Cananga odorata* (Lamk.) Hook. f. & Thomson	96	384	608
JPZ09	20161214009	Leaf	*Ceiba pentandra* (Linn.) Gaertn.	100	230	425	\	\	\	\
JPZ10	20161214010	Vine	*Dalbergia oliveri* Prain	100	218	399	\	\	\	\
JPZ11	20161214011	Bark	*Cinnamomum bejolghota* (Buch-Ham.) Sweet	100	239	438	\	\	\	\
JPZ12	20161214012	Bark	*Oroxylum indicum* (Linn.) Kurz	100	236	431	\	\	\	\
JPZ13	20161214013	Bark	*Mitragyna diversifolia* (Wall. ex G. Don) Havil	100	219	403	\	\	\	\
JPZ14	20161214014	Stem	*Abutilon indicum* (L.) Sweet	99	232	429	\	\	\	\
JPZ15	20161214015	Stem	*Allophylus longipes* Radlk.	91	243	333	Amplification failed	\	\	\
JPZ16	20161214016	Stem	*Capparis acutifolia* Sweet	83	229	147	*Capparis. mitchellii* (Lindl. ex F. Muell) Lindl.	89	422	345
JPZ17	20161214017	Stem	*Pennisetum purpureum* Schum.	99	220	401	\	\	\	\
JPZ18	20161214018	Stem	No significant similarity found	\	225	\	*C. odorata*	97	527	326
JPZ19	20161214019	Stem	*Oroxylum indicum* (Linn.) Kurz	99	236	431	\	\		
JPZ20	20161214020	Stem	*Leea guineense* G. Don	98	231	318	\	\		
JPZ21	20161214021	Root	*Puya venusta* (Baker) Phil.	95	223	353	*Ananas comosus* (Linn.) Merr.	100	564	1042
JPZ22	20161214022	Fruit	No significant similarity found	\	254	\	Amplification failed	\	\	\
JPZ23	20161214023	Stem bark	*Cinnamomum javanicum* Bl.	99	233	411	\	\	\	\
JPZ24	20161214024	Stem	*Senna alexandrina* Mill.	79	232	165	Amplification failed	\	\	\
JPZ25	20161214025	Vine	No significant similarity found	\	219	\	*Illigera rhodantha* Hance	98	425	256
JPZ26	20161214026	Stem	*Salacia agasthiamalana* Udayan, Yohannan & Pradeep	96	233	374	Amplification failed	\	\	\
JPZ27	20161214027	Stem	*Gardenia jasminoides* Ellis	94	207	309	*G. jasminoides*	100	256	464
JPZ28	20161214028	Stem bark	*Pseudoclausena chrysogyne* (Miq.) T.P. Clark	89	229	241	Amplification failed	\	\	\
JPZ29	20161214029	Stem	*Pueraria mirifica Airy Shaw & Suvat.*	93	216	154	*Spatholobus pulcher* Dunn	96	531	329
JPZ30	20161214030	Vine	*Derris trifoliata* Lour.	97	223	412	\	\	\	\
JPZ31	20161214031	Stem	*Ampelocissus martini* Planch.	92	291	418	Amplification failed	\	\	\
JPZ32	20161214032	Stem	*Pseudobaeckea teres* Dümmer	80	237	93.5	Amplification failed	\	\	\
JPZ33	20161214033	Stem	No significant similarity found	\	233	\	Amplification failed	\	\	\
JPZ34	20161214034	Root	*Croton crassifolius* Geisel.	100	204	377	\	\	\	\
JPZ35	20161214035	Stem	*Holarrhena pubescens* Wall. ex G. Don	93	238	348	Amplification failed	\	\	\
JPZ36	20161214036	Stem	*Cenchrus purpureus* (*Schumach.*) *Morrone*	100	218	403	\	\	\	\
JPZ37	20161214037	Stem	*Prismatomeris filamentosa* Craib	100	222	344	\	\	\	\
JPZ38	20161214038	Vine	*Cyphostemma trilobata* (Lam.) M.R. Almeida	96	257	453	*Cayratia trifolia* (Linn.) Domin	100	564	305
JPZ39	20161214039	Stem	*Acacia gummifera* Willd.	93	192	243	*Acacia nilotica* (Linn.) Delile	99	693	395
JPZ40	20161214040	Stem	*Erythrina subumbrans* (*Hassk.*) *Merr.*	96	234	375	*E. vespertilio* Benth.	95	399	262
JPZ41	20161214041	Stem	No significant similarity found	\	236	\	*Albizia lebbeck* (L.) Benth.	100	658	356
JPZ42	20161214042	Stem	Sequencing failed	\	\	\	Amplification failed	\	\	\
JPZ43	20161214043	Stem	Amplification failed	\	\	\	Amplification failed	\	\	\
JPZ44	20161214044	Stem	*Plumeria rubra* Linn.	86	237	204	Amplification failed	\	\	\
JPZ45	20171105001	Stem	No significant similarity found	\	202	\	*Tetracera sarmentosa* (Linn.) Vahl.	100	425	230
JPZ46	20171105002	Stem	*Salacia chinensis* L.	100	246	455	\	\	\	\
JPZ47	20171105003	Fruit	Amplification failed	\	\	\	*Pandanus tectorius* Parkinson	100	551	298
JPZ48	20171105004	Vine	*Passiflora foetida* L.	100	216	399	\	\	\	\
JPZ49	20171105005	Stem bark	*Terminalia nigrovenulosa* Pierre ex Lanessen	99	208	372	\	\	\	\
JPZ50	20171105006	Rhizomes	*Hydnophytum formicarum* Jack	99	217	357	\	\	\	\

*^1^“\” indicates that the data do not exist or the herb sample was not tested.*

Seventeen herbs could not be identified at the species level; besides the limitations of DNA barcoding, one plausible explanation can be that incomplete database information. In this context, accurate species identification by botanists is key to improve the NCBI database for identifying unknown medicinal species.

Interestingly, Chhke sreng, a pricy panacea in traditional medicine in Cambodia, was identified as Cananga brandisiana (Pierre) Saff. [syn. *Cananga latifolia* (Hook.f. & Thomson) Finet & Gagnep.] but not *Cananga odorata* (Lamk.) Hook. f. & Thomson (also called “Kdang nie”) by plant taxonomists (Dy Phon, 2000).

However, *C. odorata* and Chhke sreng are related to each other as indicated by several references, presumably owing to the mismatch of local and Latin names of some medicinal species or the adulteration and mis-authentication of *C. odorata* in Cambodian markets. Thus, DNA barcoding plays a key role in ensuring medicinal safety in Cambodia and it would be better when DNA barcoding combines chemical information.

**FIGURE 2:**
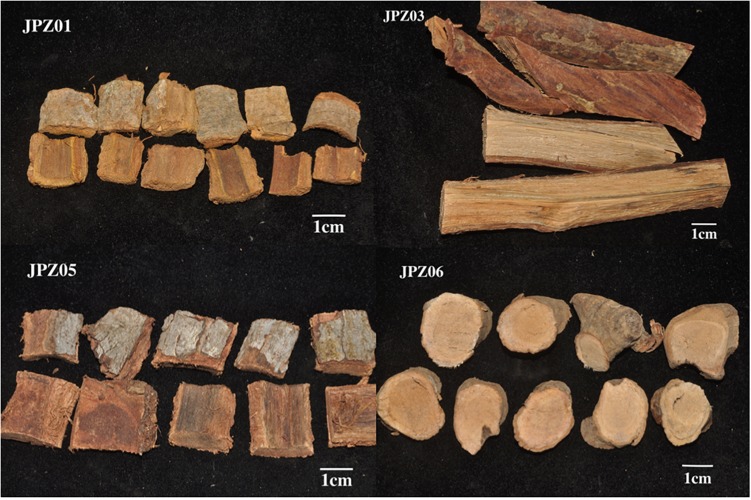
Representative medicinal herbs obtained from a domestic market.

The ethnopharmacological information (family name, distribution, local name) of 33 plants is shown in Table 2. Among these, there were five Rubiaceae and eight Fabaceae plants. The pictures of five representative medicines are shown in Figure 2. Furthermore, legumes were the most cited plants in studies from Cambodia, Thailand, and Laos (Chassagne et al., 2017).

**TABLE 2:** Ethnopharmacological information for the 33 medicinal plants of defined species.

Number	Identification	Family name	Distribution	Local name
JPZ01	*Nauclea officinalis* (Pierre ex Pit.) Merr. & Chun	Rubiaceae	China, Borneo, Cambodia, Indonesia (Sumatra), Laos, Malaysia, Philippines, Thailand, Vietnam.	Ktum tuk
JPZ02	*Ficus sagittata* Vahl	Moraceae	China, Bhutan, India, Indonesia, Myanmar, Philippines, Sikkim, Thailand, Vietnam, Cambodia.	Krabei Leung Kor (Dy Phon, 2000)
JPZ03	*Dalbergia pinnata* (Lour.) Prain	Fabaceae	China, Indonesia (Java), Laos, Malaysia, Myanmar, Philippines, Thailand, Vietnam.	Unknown
JPZ04	*Flacourtia indica* (Burm.f.) Merr.	Flacourtiaceae	Widespread and cultivated in tropical and subtropical regions of Africa, Asia, the Pacific Islands.	Krorkob (Prey) (Turreira-García et al., 2017)
JPZ05	*Anacardium occidentale* L.	Anacardiaceae	Native to tropical America, now widely cultivated in the global tropics.	Dio/Savy Chanti
JPZ06	*Calamus acanthospathus* Griff.	Arecaceae	China, Vietnam, Laos, Thailand, India.	Pdao (Dy Phon, 2000)
JPZ09	*Ceiba pentandra* (L.) Gaertn.	Bombacaceae	Native to tropical America and possibly West Africa, now pantropical.	Ko
JPZ10	*Dalbergia oliveri* Prain	Fabaceae	Myanmar, Thailand, Laos, Cambodia, Vietnam.	Niang Nuan
JPZ11	*Cinnamomum bejolghota* (Buch.-Ham.) Sweet	Lauraceae	China, Bangladesh, Bhutan, India, Laos, Myanmar Nepal, Thailand, Vietnam, Cambodia.	Teppiroo (Turreira-García et al., 2017)
JPZ12/19	*Oroxylum indicum* (L.) Kurz	Bignoniaceae	Bhutan, Cambodia, India, Indonesia (Java, Sumatra), Laos, Malaysia, Myanmar, Nepal, Philippines, Thailand, Vietnam.	Pou Long
JPZ13	*Mitragyna diversifolia* (Wall. ex G.Don) Havil.	Rubiaceae	China, Cambodia, Indonesia, Laos, Malaysia, Myanmar, Philippines, Thailand, Vietnam.	Unknown
JPZ14	*Abutilon indicum* (L.) Sweet	Malvaceae	China, Bhutan, Cambodia, India, Indonesia, Laos, Myanmar, Nepal, Sri Lanka, Thailand, Vietnam.	Tbal Kenn
JPZ17	*Cenchrus purpureus (Schumach.) Morrone*	Poaceae	Native to Africa. Introduced and cultivated to India, Myanmar, Oceania, the Americas.	Smao Kantuy Chhker (Dy Phon, 2000)
JPZ18	*Cananga odorata* (Lam.) Hook.f. & Thomson	Annonaceae	Native to NE Australia, India, Indonesia, Laos, Malaysia, Myanmar, Philippines, Thailand.	Chhke Sreng (PlantUse English,, 2018) (*Cananga odorata*) (Materia Aromatica,, 2019)
JPZ20	*Leea guineensis* G.Don	Leeaceae	Bangladesh, Bhutan, Cambodia, India, Indonesia, Laos, Malaysia, Myanmar, Nepal, New Guinea, Philippines, Thailand, Vietnam, Africa, Madagascar.	Kdaing Bay (Dy Phon, 2000)
JPZ21	*Ananas comosus* (L.) Merr.	Bromeliaceae	Native to the American tropics; the cultivated pineapples are grown mainly between latitudes 24°N and 25°S.	PritNgo/NgoBone
JPZ23	*Cinnamomum javanicum* Blume	Lauraceae	From southern China to Peninsular Malaysia, Sumatra, Java, Borneo.	Krorvanh deum (Dy Phon, 2000)
JPZ25	*Illigera rhodantha* Hance	Hernandiaceae	China, Cambodia, Laos, Thailand, Vietnam.	Vor Kroch (Dy Phon, 2000)
JPZ27	*Gardenia jasminoides* J.Ellis	Rubiaceae	China, Bhutan, Cambodia, India, Japan, North Korea, Laos, Nepal, Pakistan, Thailand, Vietnam; cultivated in Africa, Asia, Australia, Europe, North and South America, Pacific Islands.	Unknown
JPZ30	*Derris trifoliata* Lour.	Fabaceae	China, Cambodia, India, Indonesia, Japan, Malaysia, Papua New Guinea, Sri Lanka, Thailand, Vietnam, East Africa, Australia, Pacific Islands.	Vor Breng Krohom (Dy Phon, 2000)
JPZ34	*Croton crassifolius* Geiseler	Euphorbiaceae	China, Laos, Myanmar, Thailand, Vietnam, Cambodia.	Bongki or Pongki (Dy Phon, 2000)
JPZ36	*Lablab purpureus* (L.) Sweet	Fabaceae	Native to Africa; cultivated throughout the tropics.	Sandek Baraing
JPZ37	*Prismatomeris filamentosa* Craib	Rubiaceae	China, Cambodia, India, Thailand, Vietnam.	Romdenhmeas
JPZ38	*Cayratia trifolia* (L.) Mabb. & J.Wen	Vitaceae	China, Bangladesh, Cambodia, India, Indonesia, Laos, Malaysia, Nepal, Thailand, Vietnam.	Tror Det
JPZ39	*Acacia nilotica* (L.) Delile	Leguminosae	Native to Africa and extends to Arabia, Afghanistan, India; now cultivated in many parts of the world.	Unknown
JPZ41	*Albizia lebbeck* (L.) Benth.	Fabaceae	Native to tropical Africa; introduced or naturalized in Bangladesh, Bhutan, India, Myanmar, Nepal, Pakistan, Sri Lanka, Cambodia.	Daem Chrees
JPZ45	*Tetracera sarmentosa* (L.) Vahl	Dilleniaceae	China, India, Indonesia, Malaysia, Myanmar, Sri Lanka, Thailand, Cambodia.	Daskorn (Dy Phon, 2000)
JPZ46	*Salacia chinensis* L.	Celastraceae	China, Cambodia, India, Indonesia, Laos, Malaysia, Myanmar, Philippines, Sri Lanka, Thailand, Vietnam.	Veay (Chassagne et al., 2017)
JPZ47	*Pandanus tectorius* Parkinson ex Du Roi	Pandanaceae	China, Southeast Asia, tropical Australia, Pacific Islands (Polynesia).	Romchek Srok (Dy Phon, 2000)
JPZ48	*Passiflora foetida* L.	Passifloraceae	Native to the southwestern United States, Mexico, the Caribbean, Central America, and much of South America; prevalent in tropical regions around the world.	Sao Mao Prey
JPZ49	*Terminalia nigrovenulosa* Pierre	Combretaceae	China, Cambodia, Laos, Malaysia, Myanmar, Thailand, Vietnam.	Bayarm
JPZ50	*Hydnophytum formicarum* Jack	Rubiaceae	Cambodia	Sourt Damrey (Keo et al., 2018)

Importantly, 19 out of 33 medicinal plants were also used as Chinese medicines, and they were *Nauclea officinalis*, *Ficus sagittata*, *Dalbergia pinnata*, *Flacourtia indica*, *Anacardium occidentale*, *Dalbergia oliveri*, *Cinnamomum bejolghota*, *Oroxylum indicum*, *Abutilon indicum*, *Illigera rhodantha*, *Gardenia jasminoides*, *Derris trifoliata*, *Croton crassifolius*, *Lablab purpureus*, *Cayratia trifolia*, *Acacia nilotica*, *Pandanus tectorius*, *Passiflora foetida*, and *Terminalia nigrovenulosa.* Among these, *O. indicum*, *P. tectorius*, and *N. officinalis* were the typical traditional Chinese herbs with a long history of use. *N. officinalis*, also known as Li minority medicine from which several new drugs have been developed, can lower body temperature, detoxify the body, reduce swelling, and relieve pain. It is often used in the treatment of colds, fevers, acute tonsillitis, pharyngitis, pneumonia, urinary system infections, enteritis, dysentery, carbuncle, and other diseases. *Oroxylum indicum* has been included in the 2015 Chinese Pharmacopoeia for treating fevers, coughs, sore throats, lung phlegm, liver diseases, and stomach pain (Chinese Pharmacopoeia Comission, 2015). The flowers, leaves, fruits, roots, and rhizomes of *P. tectorius* have high medicinal value in the remedy of colds, fevers, hepatitis, dysentery, hemorrhoids, orchitis, syphilis, and diabetes mellitus (Zhan, 2013). Therefore, studies are needed to further characterize these plants of medicinal value. According to the results shown in Table 2, there were 19 species distributed in Guangdong, 17 species in Guangxi, and 15 species in Yunnan. Therefore, the medicinal plants in Cambodia are similar to the three aforementioned provinces in China.

## Materials and Methods

### Study Area and Materials

In Orussey Market, we interviewed a total of 10 medicine retailors, all sellers were briefed on the purpose and details of the investigation, they were also informed that the investigation could be terminated any time as needed. As shown in Tables 1, 2, we collected medicinal plant samples and recorded the local names of the medicinal plants. Some local names of these plants were provided by the sellers, others were obtained by reviewing the literature. Ethnopharmacological information including family name and distribution of medicinal species were also obtained from the literature. The medicinal material samples were dried and stored in Herbarium of Institute of Medicinal Plant Development, Chinese Academy of Medical Sciences (IMD). Voucher numbers are listed in Table 1.

### DNA Extraction

Approximately 30 mg of each sample was ground for 2 min (40 Hz) using a high-throughput tissue grinder (Scientz-48, Ningbo, China). Total genomic DNA was extracted using a plant genomic DNA extraction kit (Tiangen Biotech Co., China). Occasionally, alien DNA sequences from other species – such as fungi and algae – or mixed sequence signals were repeatedly detected if the primers were not specific. To prevent non-specific PCR amplification, we washed the samples of medicinal materials using 75% alcohol to remove fungi and other plant powder contaminations.

### PCR Amplification and Sequencing

The primers used for amplification and sequencing were as follows: ITS2 (the second ITS) (forward, 5′-GCGATACTTG GTGTGAAT-3′; reverse, 5′-GACGCTTCTCCAGACTACA AT-3′) (Chen et al., 2010) and *psb*A-*trn*H intergenic spacer [forward, 5′-GTTATGCATGAACGTAATGCTC-3′ (Sang et al., 1997); reverse, 5′-CGCGCATG GTGGATTCACAATCC-3′ (Tate and Simpson, 2003)]. Primers were synthesized by Shanghai Shenggong Bioengineering Co., Ltd. The 25 μL PCR reaction contained 12.5 μL of 2× *Taq* PCR Mix, 1.0 μL each of the forward and reverse primers (2.5 μmol L^–1^), 8.5 μL of double distilled water, and 2.0 μL of the template (genomic DNA < 0.1 ng). The PCR amplification procedure for ITS2 was as follows: denaturation at 94°C for 5 min, followed by 40 cycles of denaturation at 94°C for 30 s, annealing at 56°C for 30 s, and extension at 72°C for 45 s. The PCR amplification procedure for *psb*A-*trn*H was as follows: denaturation at 95°C for 4 min, followed by 35 cycles of denaturation at 94°C for 30 s, annealing at 55°C for 1 min, and extension at 72°C for 1 min. A final extension was performed at 72°C for 10 min for both PCR amplification procedures. The PCR was conducted in a thermal cycler (model 2720; Thermo Fisher Scientific). Bi-directional sequencing of the PCR products was performed by Beijing Qingke New Industry Biotechnology Co., Ltd.

### Data Analysis

Codon Code Aligner V 7.0.1 (CodonCode Co., United States) was used to assemble and cut the contigs and to generate the ITS2 and *psb*A-*trn*H sequences. The sequences were submitted to the National Center for Biotechnology Information (NCBI) database to search for other similar sequences, which have been taxonomically validated from published literatures. To identify the species of each medicinal plant, each species was searched in the literature in descending order of similarity. The maximum score was used to determine if the medicinal plant distributed in Cambodia. If its origin was from Cambodia and its similarity was ≥97% [97% was used as the DNA barcoding identification similarity threshold for medicinal plants (Chen et al., 2012; Gu et al., 2015)], the species identified were considered to be the final. If the similarity was ≤97%, the *psb*A-*trn*H sequence was amplified. The final identification results of similarities ≥97% were determined according to the aforementioned ITS2 method. If the similarity was between 90 and 97%, the results revealed the genus level. In cases of inconsistent ITS2 and *psb*A-*trn*H results, we choose the one with the higher similarity.

### Ethics

We plan to work with the National Center of Traditional Medicine (NCTM), Ministry of Health of Cambodia, to publish *Handbook of medicinal plants in Cambodia*, which will be made available to the Cambodian sellers we interviewed after its publication. Meanwhile, we also collaborate with NCTM on the Sino-Cambodian International Exchange Project to promote educational and academic communications between China and Cambodia; this project will also provide Cambodia with technical and theoretical supports in the identification and marker-assisted selection of medicinal plants.

## Conclusion

Cambodia has a history of nearly 2000 years, and for a long time, it has suffered from civil wars and wars of aggression. In light of the extremely poor conditions, the Cambodian people have relied on their own practices to identify medicinal plants to fight diseases. Although there is some information on various medicinal plants, there is no pharmacopoeia and no readily available body of medicinal plant literature, which has hindered the application of medicinal plants and the dissemination of results from investigators of other countries. Therefore, the current study of medicinal plants in Cambodia is incomplete, and there remain many gaps in knowledge. Furthermore, many plants have become endangered due to industrialization and environmental pollution (Chassagne et al., 2016). In light of this, the DNA barcoding technique can provide useful information on the species of various medicinal plants in Cambodia. This will not only preserve plant knowledge in Cambodia, but also help develop an ethnopharmacopia and provide new insights on the development of new drugs in the future. At the same time, the use of DNA barcoding is one step in supporting the improvement in the quality control of plants being sold for medicinal use in Cambodia and it will emphasize the importance of protecting endangered species. This approach can also be used in other countries or regions with relatively backward economies and underdeveloped research practices. Through DNA barcoding, commonly used medicinal plants can be completely characterized.

## Data Availability Statement

All datasets generated for this study are included in the article/Supplementary Material.

## Author Contributions

XC, XZ, WL, YQ, TH, RL, and YY: investigation. WS, XZ, YS, XM, and YM: methodology. JW and XZ: project administration. XZ, WS, and WL: resources. JW: supervision. XC: writing – original draft. XZ and WS: writing – review and editing.

## Conflict of Interest

The authors declare that the research was conducted in the absence of any commercial or financial relationships that could be construed as a potential conflict of interest.
