# Supplementary figures and images for: Assessing the Identity of Commercial Herbs From a Cambodian Market Using DNA Barcoding
Source: Front Pharmacol. 2020 Mar 24;11:244. doi: 10.3389/fphar.2020.00244 (PMC7105672; doi:10.3389/fphar.2020.00244)

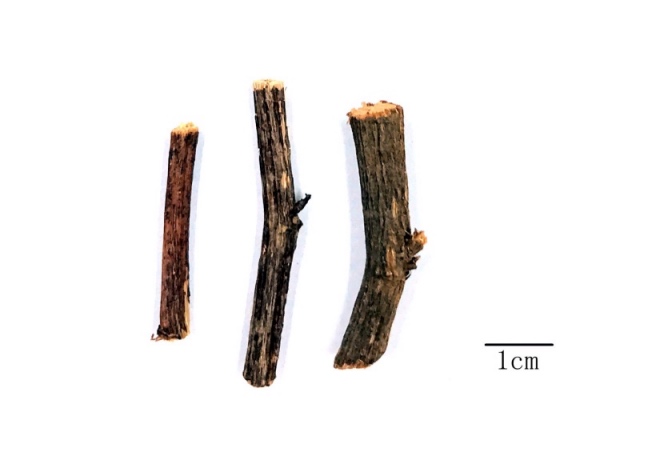

Supplement: Supplementary file 3 [file Data_Sheet_3.zip › Fotor Batch/JPZ49.jpg]

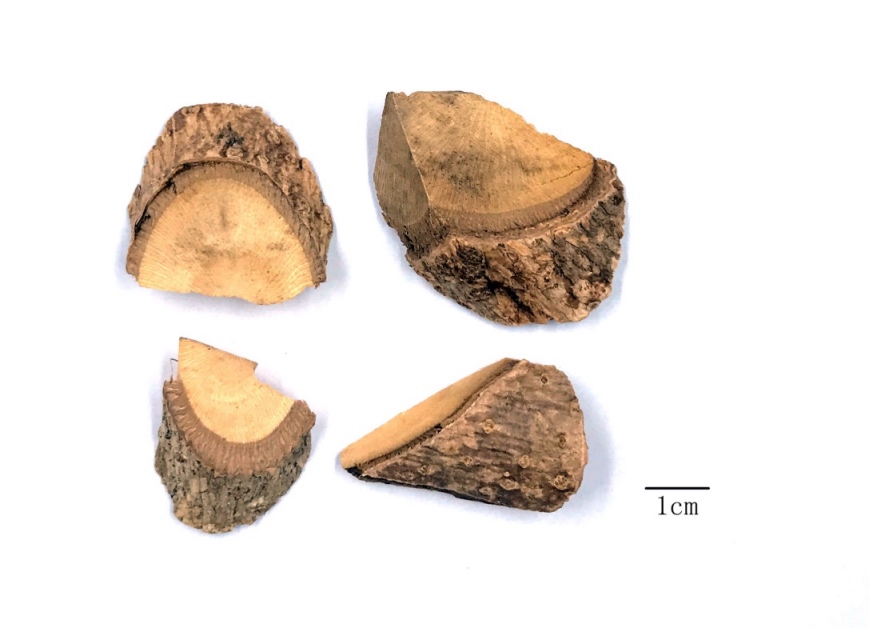

Supplement: Supplementary file 3 [file Data_Sheet_3.zip › Fotor Batch/JPZ48.jpg]

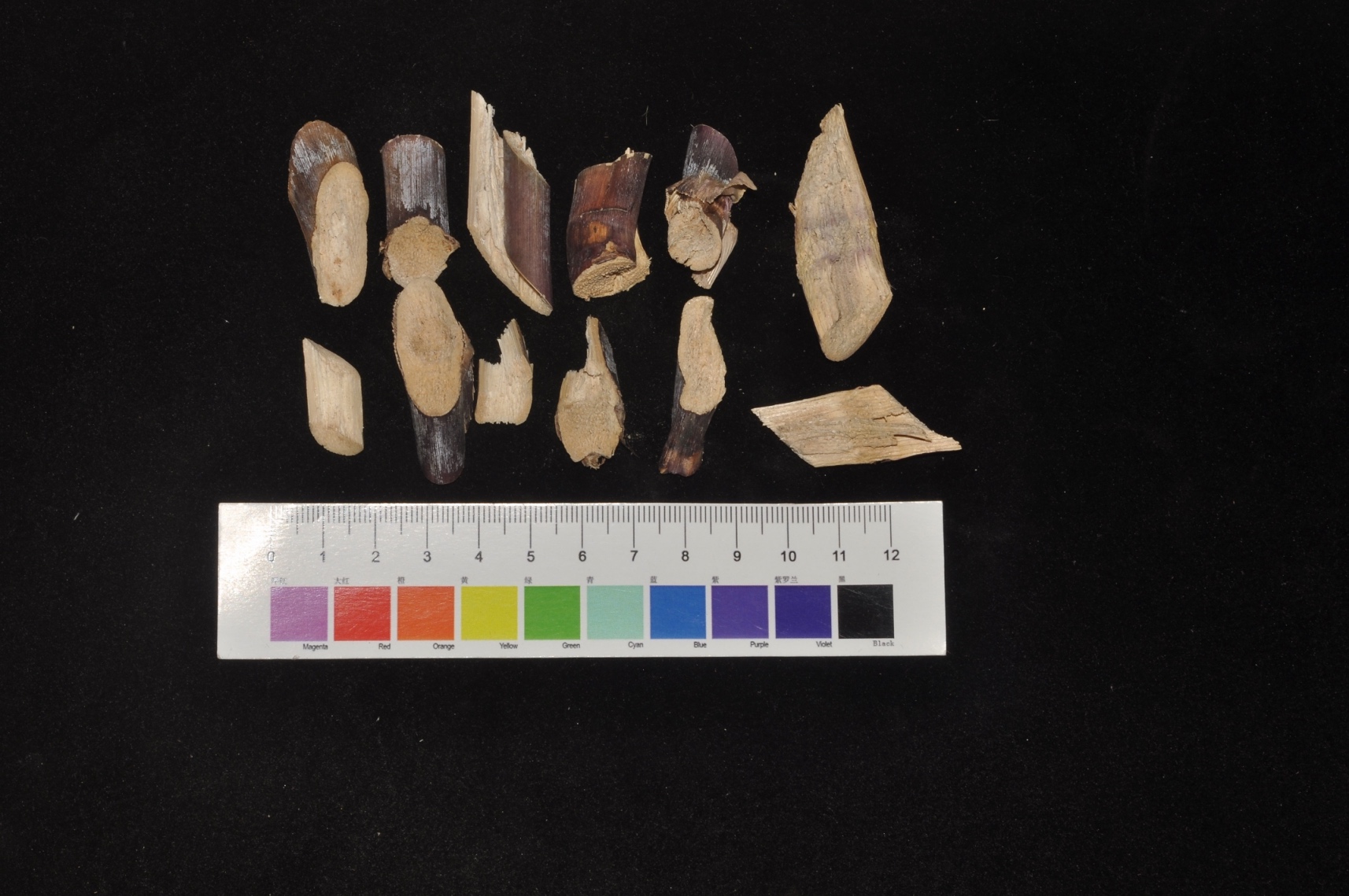

Supplement: Supplementary file 3 [file Data_Sheet_3.zip › Fotor Batch/JPZ16.jpg]

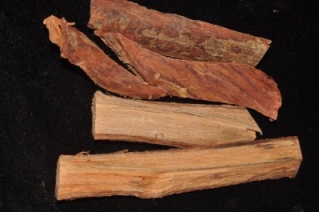

Supplement: Supplementary file 3 [file Data_Sheet_3.zip › Fotor Batch/JPZ02.jpg]

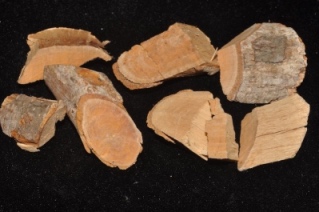

Supplement: Supplementary file 3 [file Data_Sheet_3.zip › Fotor Batch/JPZ03.jpg]

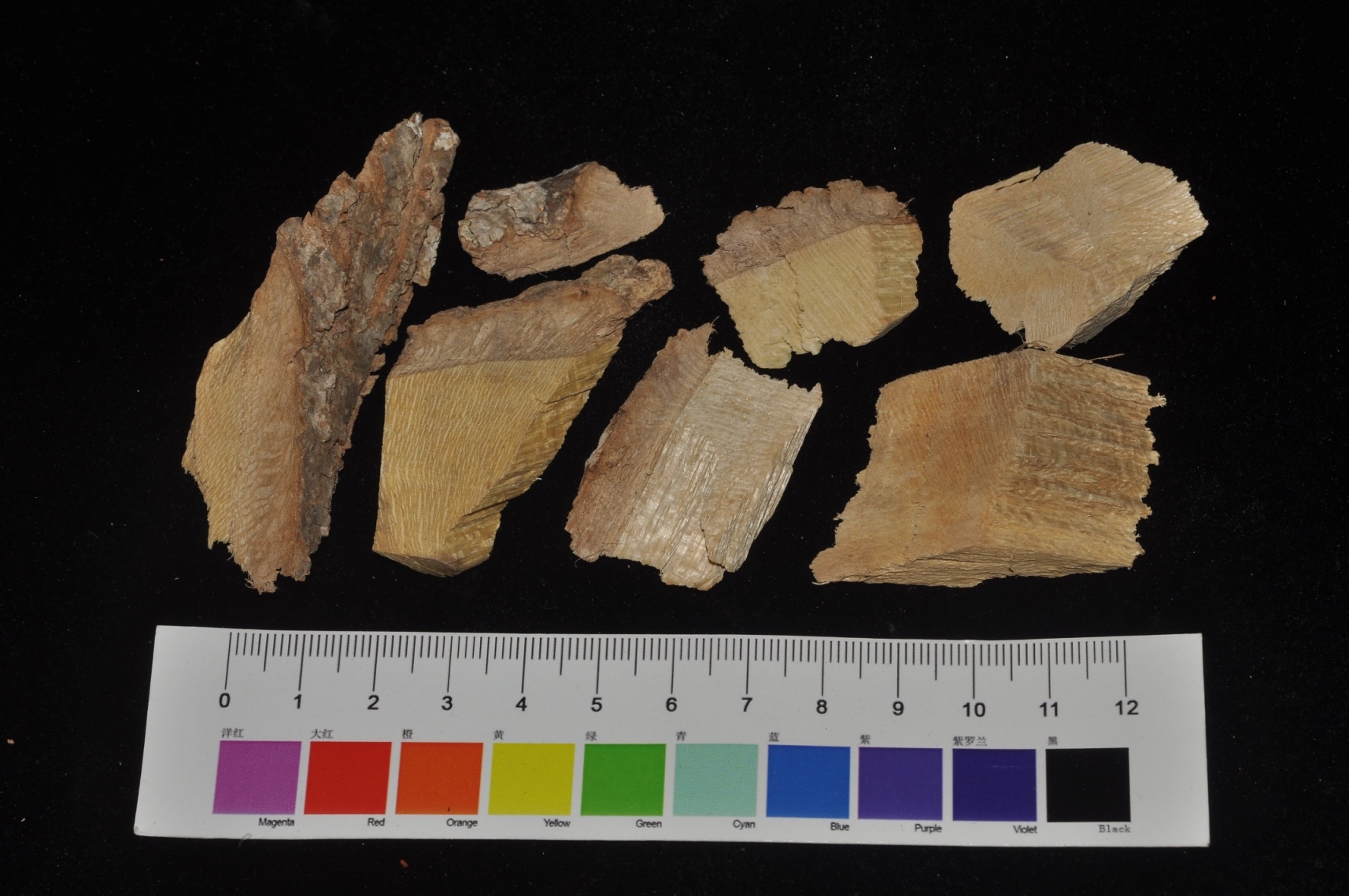

Supplement: Supplementary file 3 [file Data_Sheet_3.zip › Fotor Batch/JPZ17.jpg]

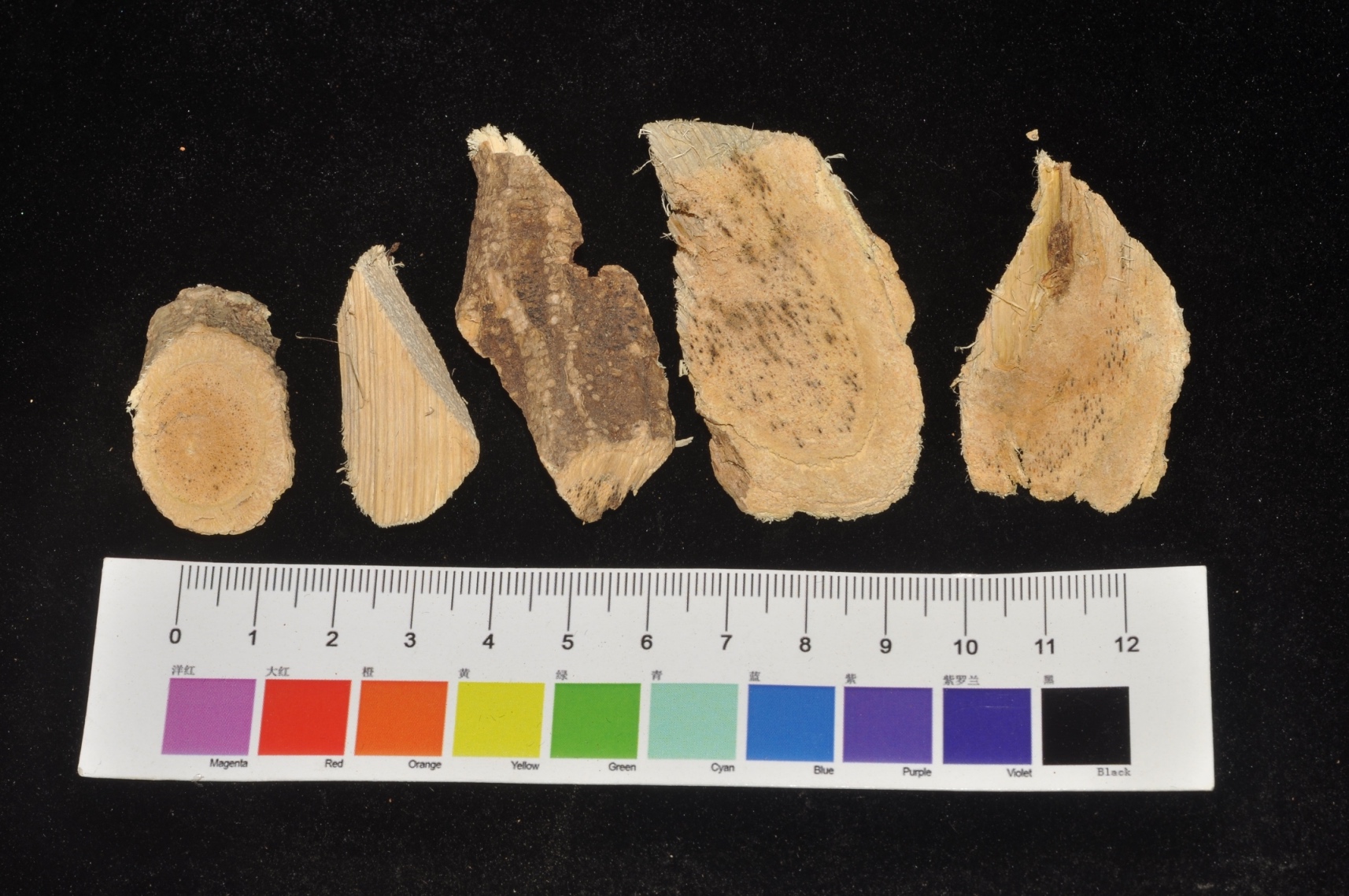

Supplement: Supplementary file 3 [file Data_Sheet_3.zip › Fotor Batch/JPZ29.jpg]

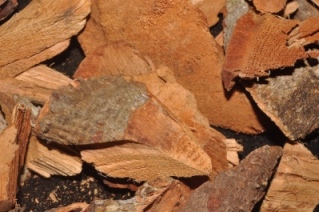

Supplement: Supplementary file 3 [file Data_Sheet_3.zip › Fotor Batch/JPZ01.jpg]

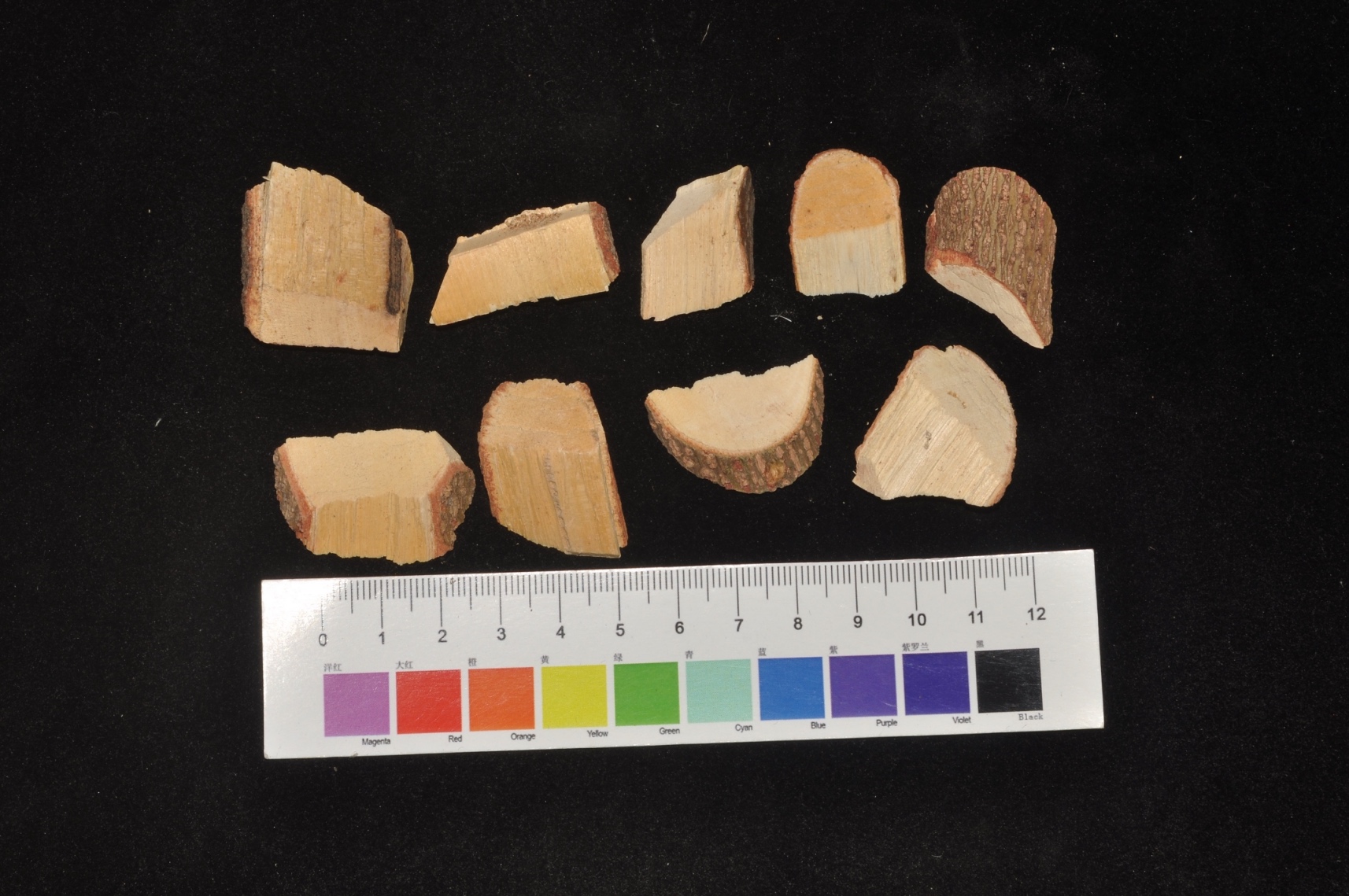

Supplement: Supplementary file 3 [file Data_Sheet_3.zip › Fotor Batch/JPZ15.jpg]

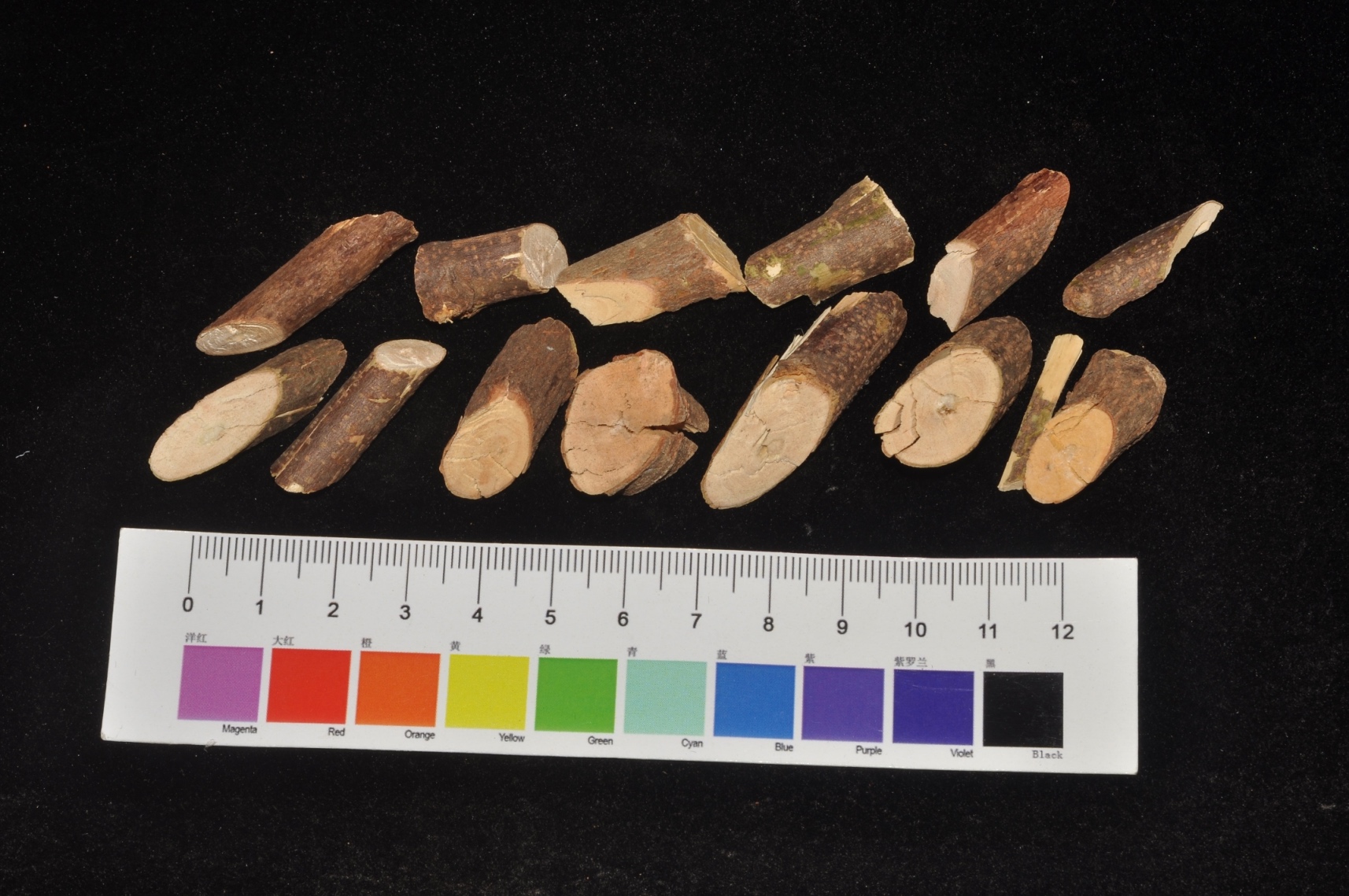

Supplement: Supplementary file 3 [file Data_Sheet_3.zip › Fotor Batch/JPZ14.jpg]

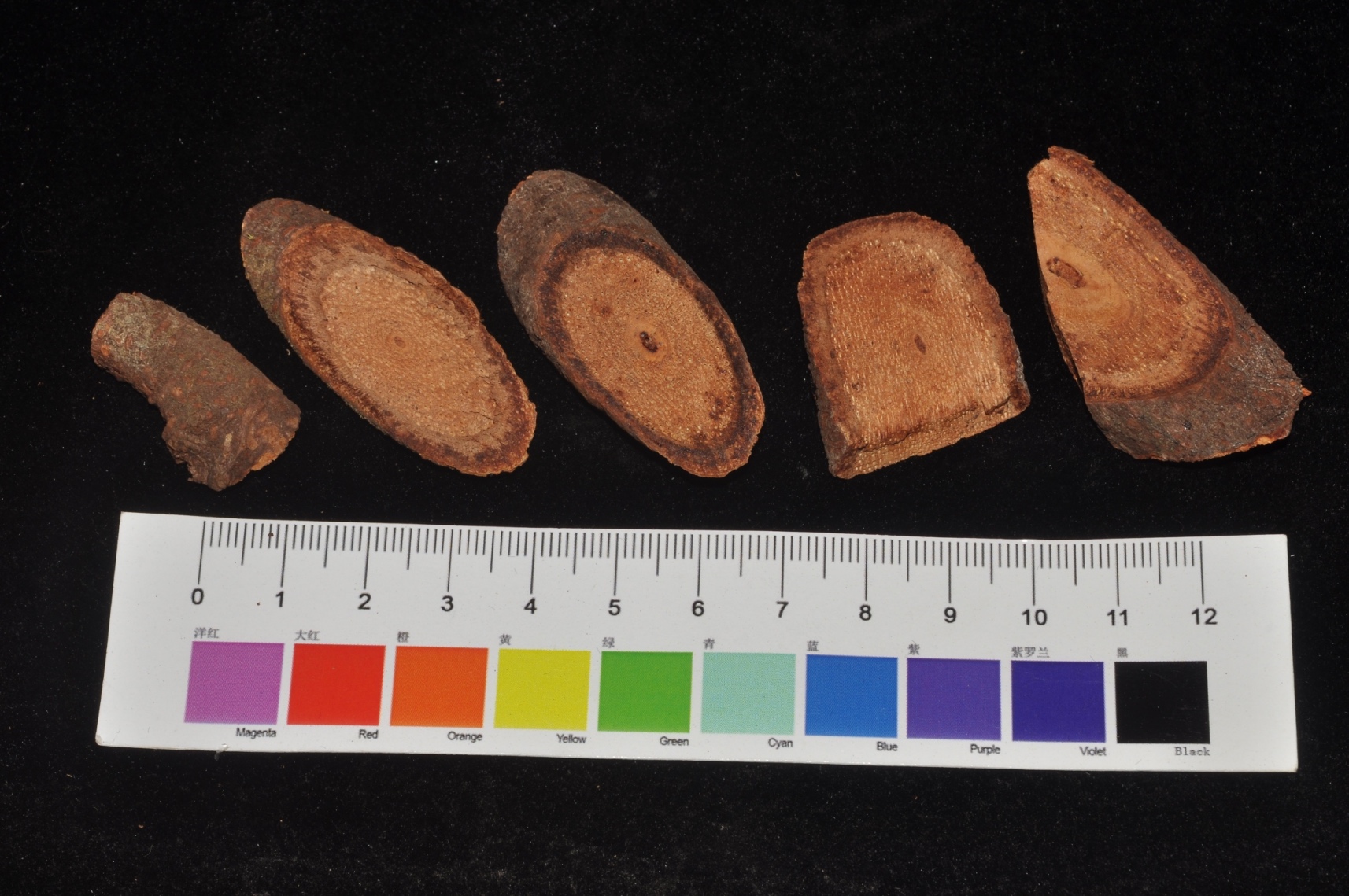

Supplement: Supplementary file 3 [file Data_Sheet_3.zip › Fotor Batch/JPZ28.jpg]

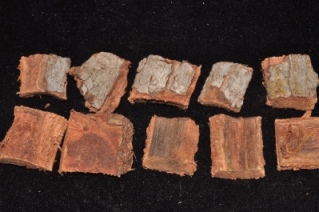

Supplement: Supplementary file 3 [file Data_Sheet_3.zip › Fotor Batch/JPZ04.jpg]

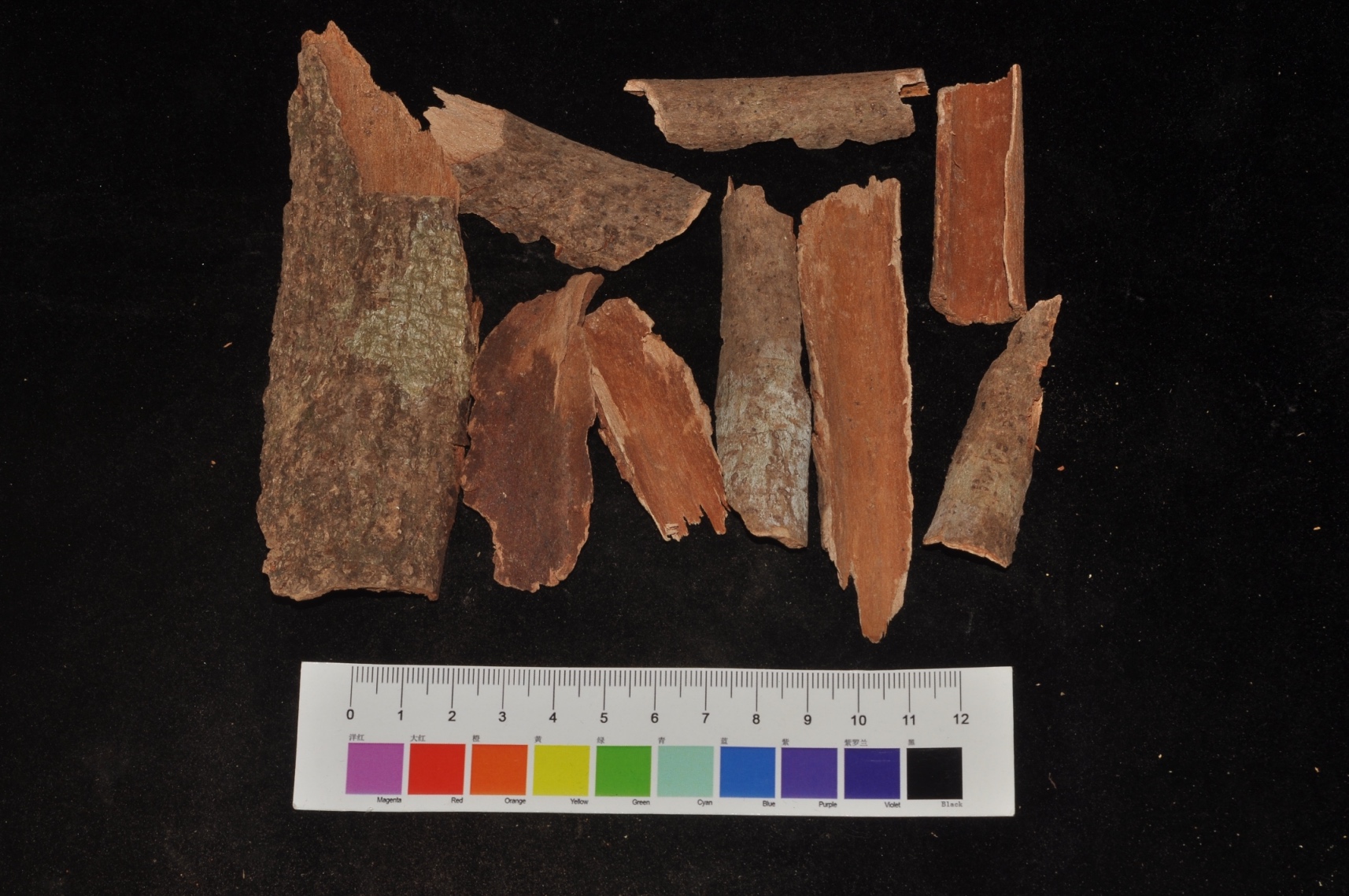

Supplement: Supplementary file 3 [file Data_Sheet_3.zip › Fotor Batch/JPZ10.jpg]

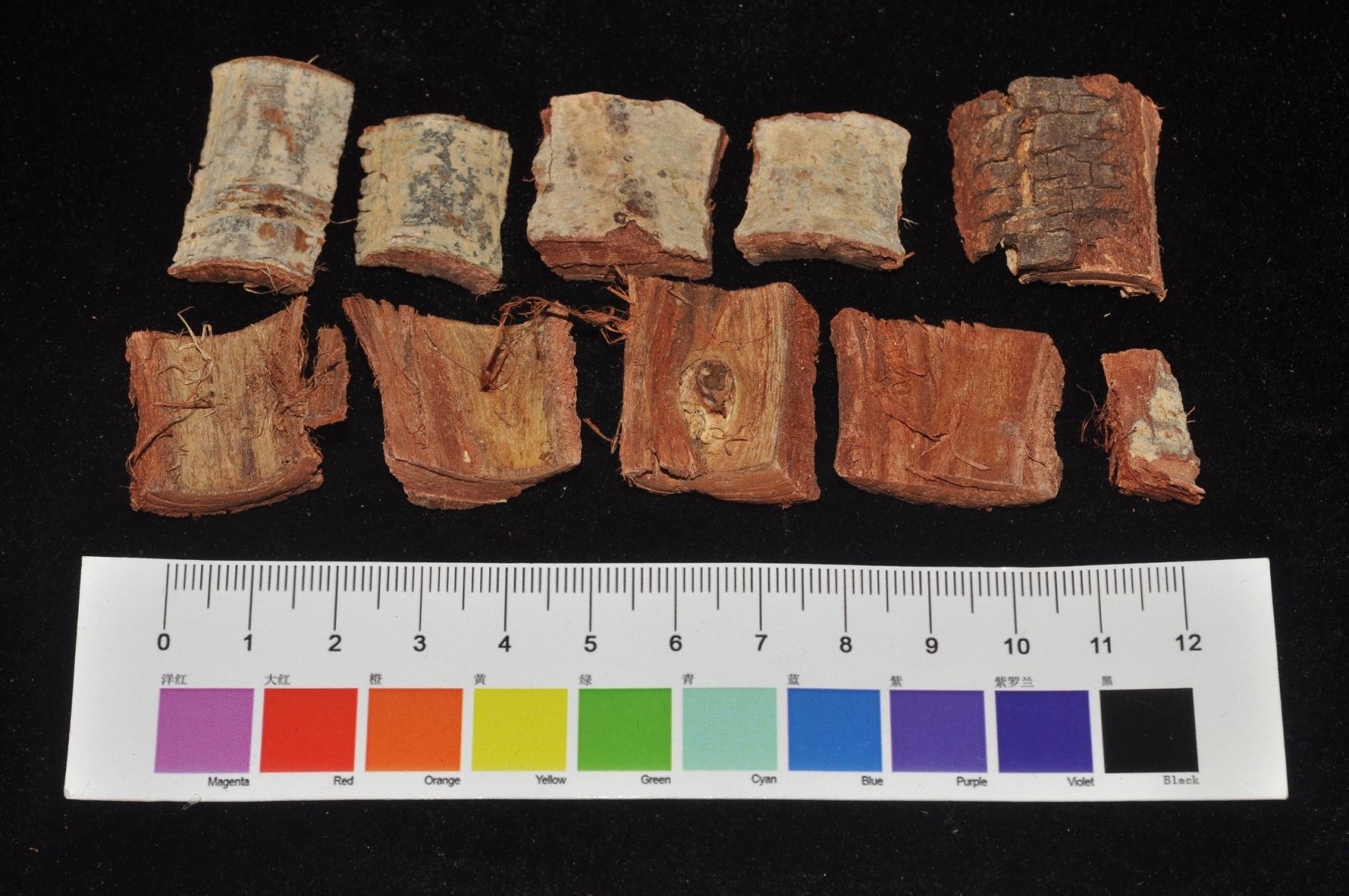

Supplement: Supplementary file 3 [file Data_Sheet_3.zip › Fotor Batch/JPZ38.jpg]

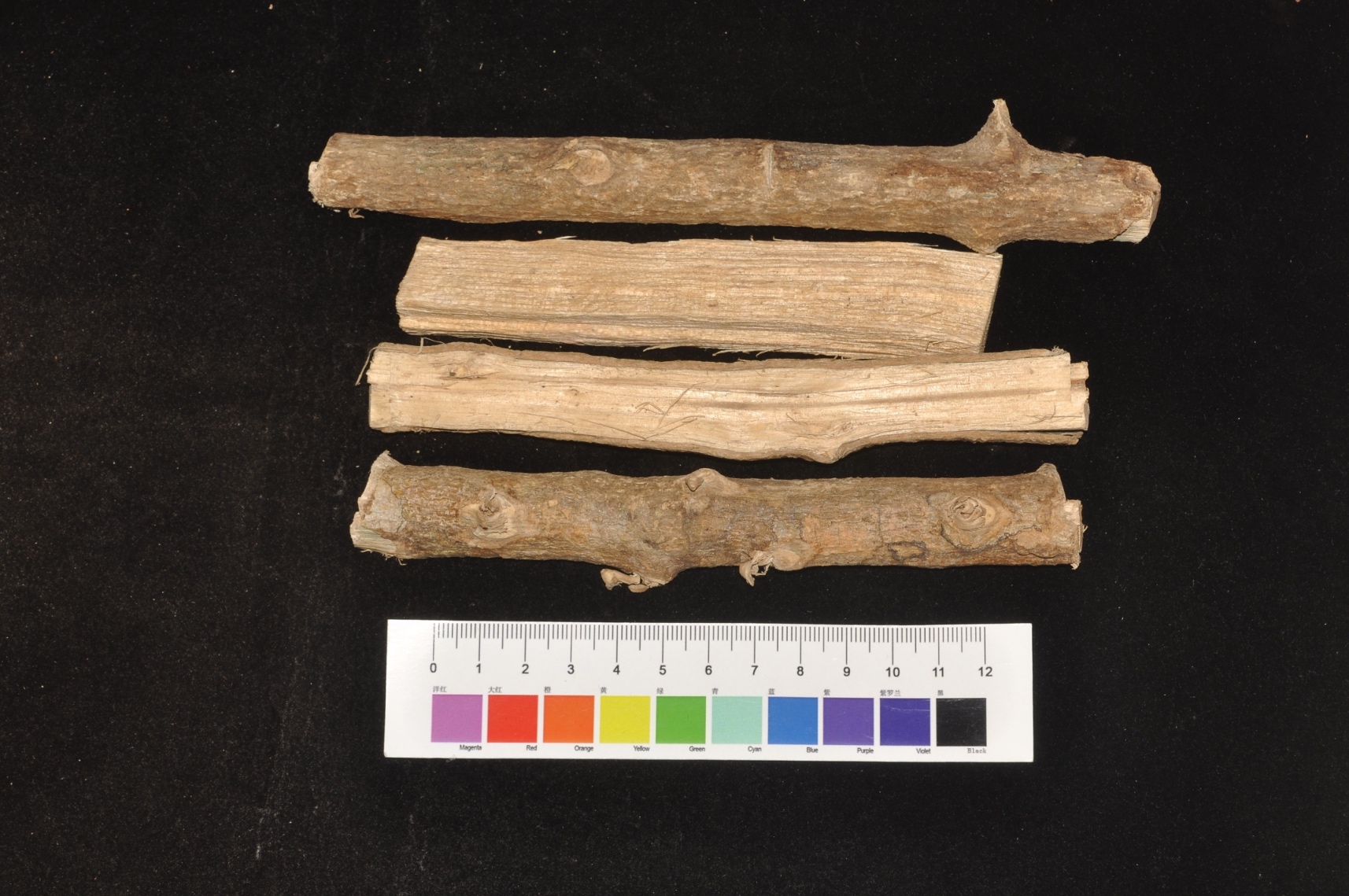

Supplement: Supplementary file 3 [file Data_Sheet_3.zip › Fotor Batch/JPZ39.jpg]

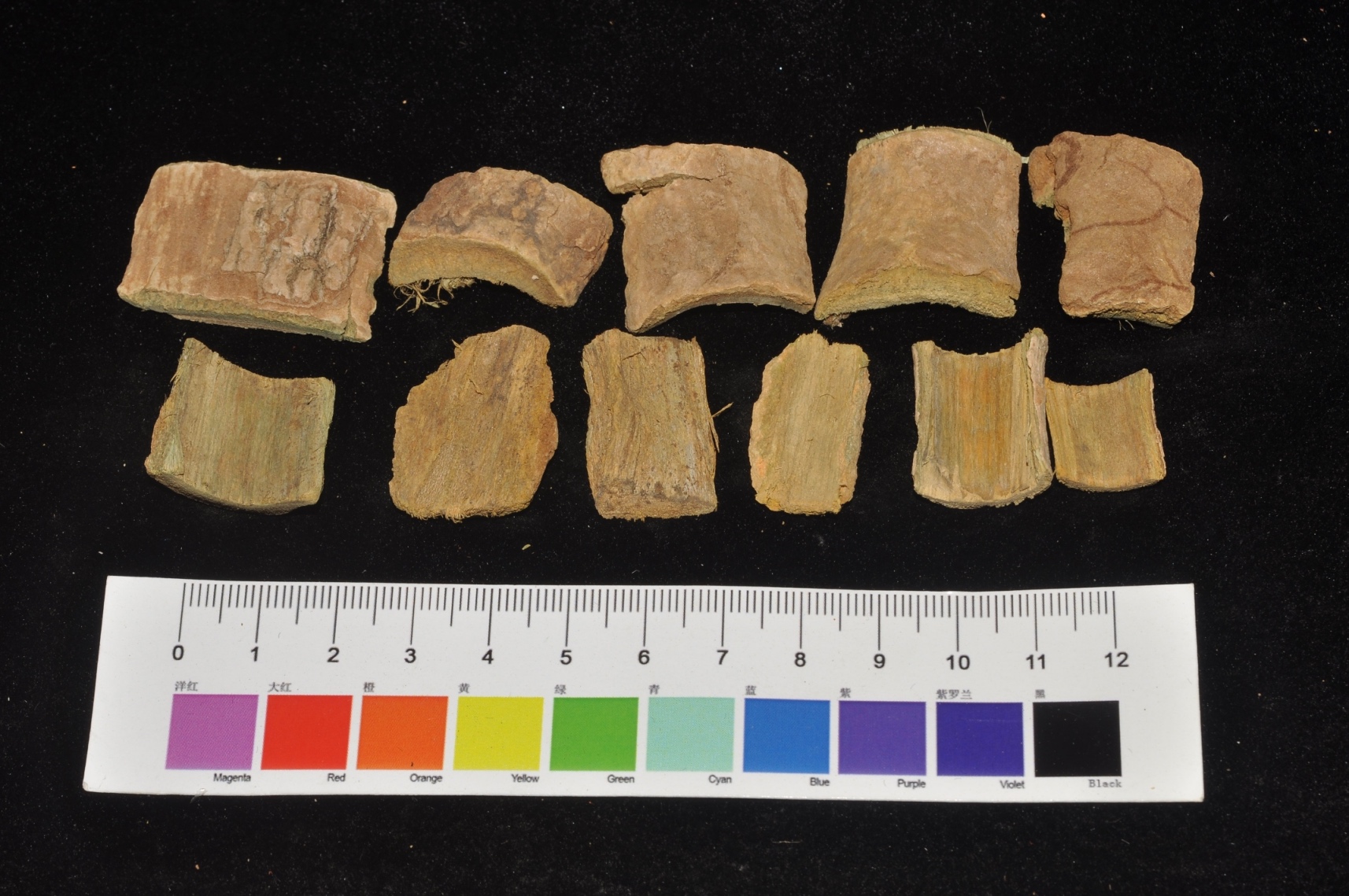

Supplement: Supplementary file 3 [file Data_Sheet_3.zip › Fotor Batch/JPZ11.jpg]

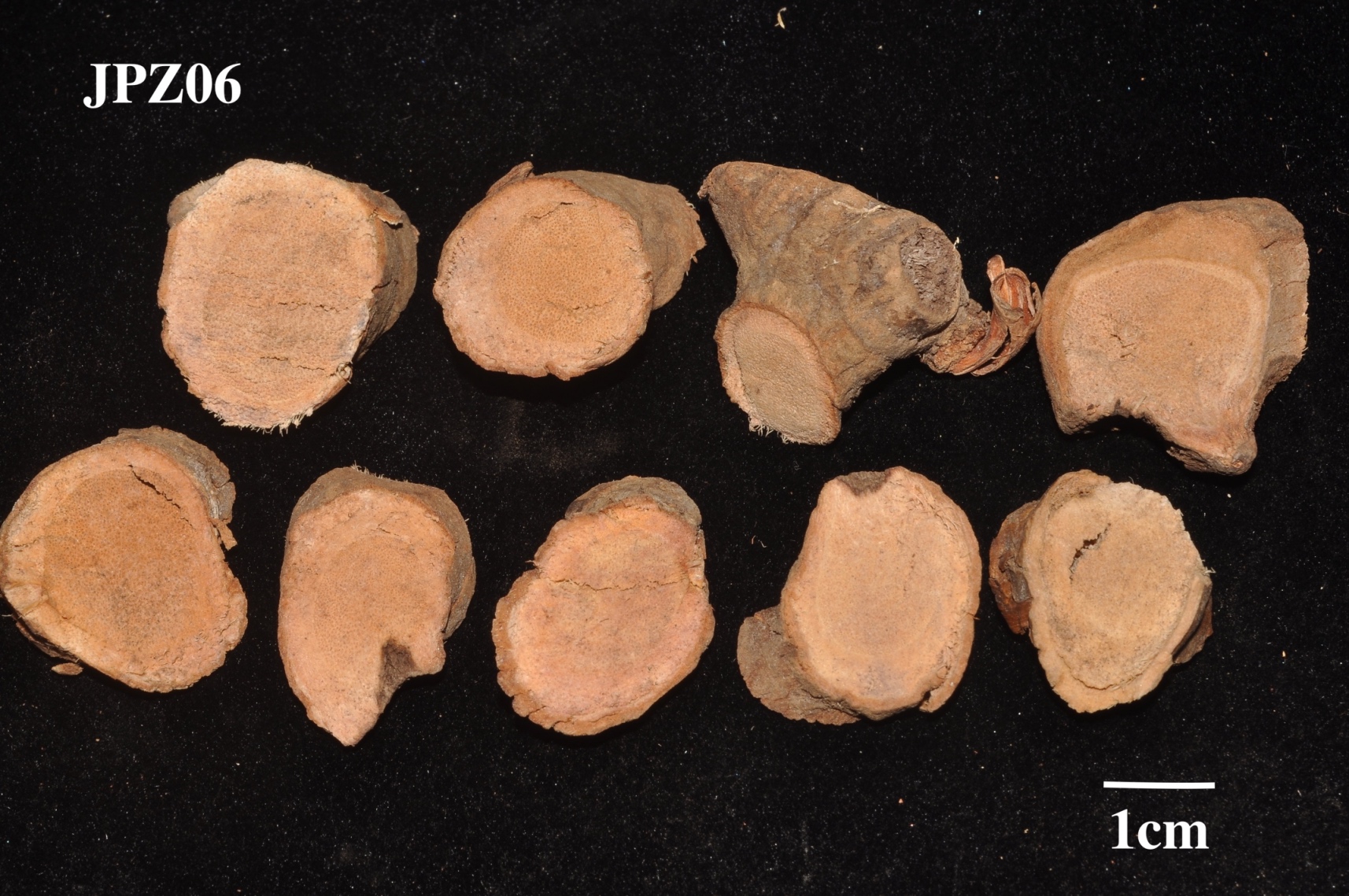

Supplement: Supplementary file 3 [file Data_Sheet_3.zip › Fotor Batch/JPZ05.jpg]

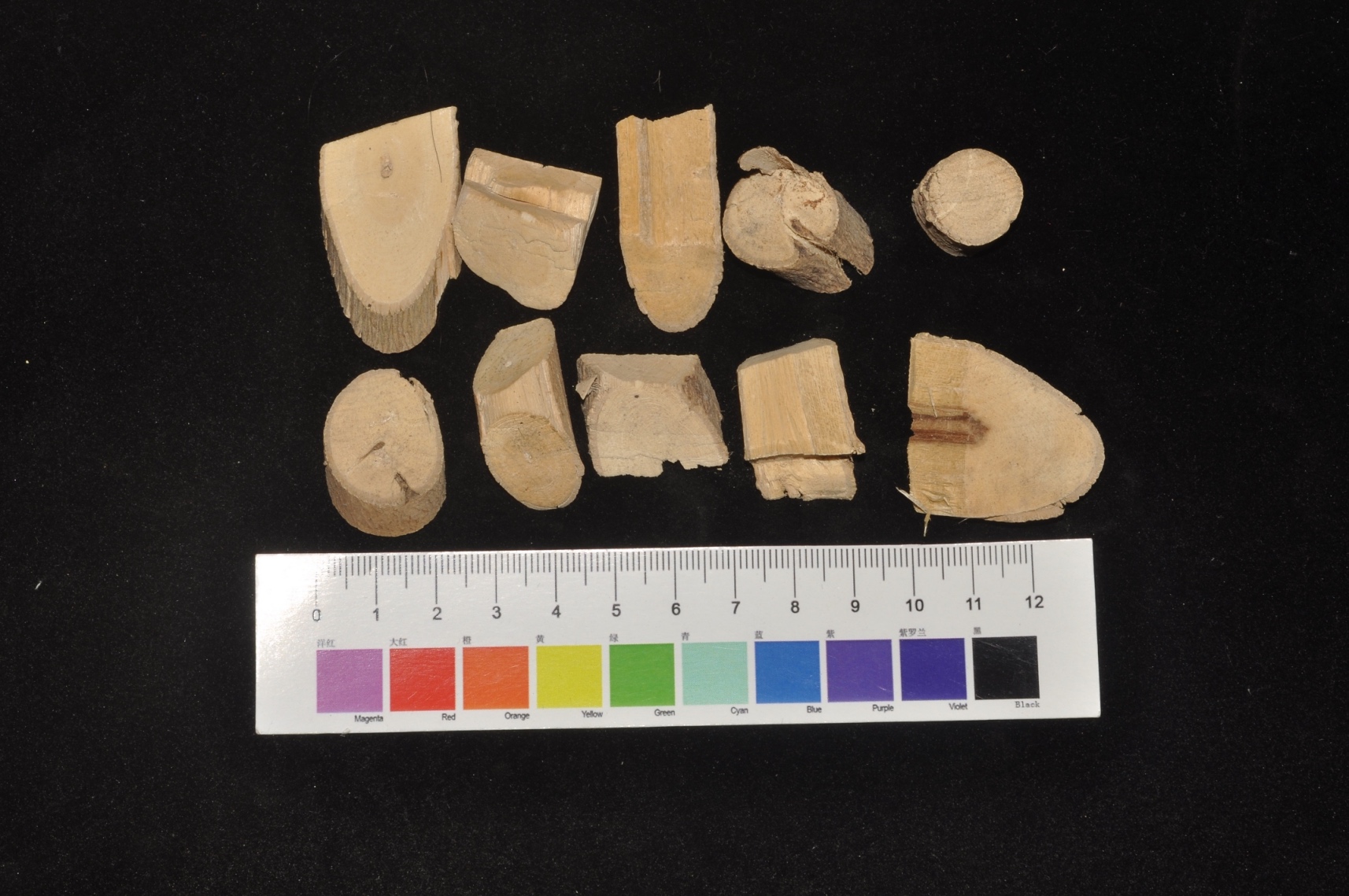

Supplement: Supplementary file 3 [file Data_Sheet_3.zip › Fotor Batch/JPZ13.jpg]

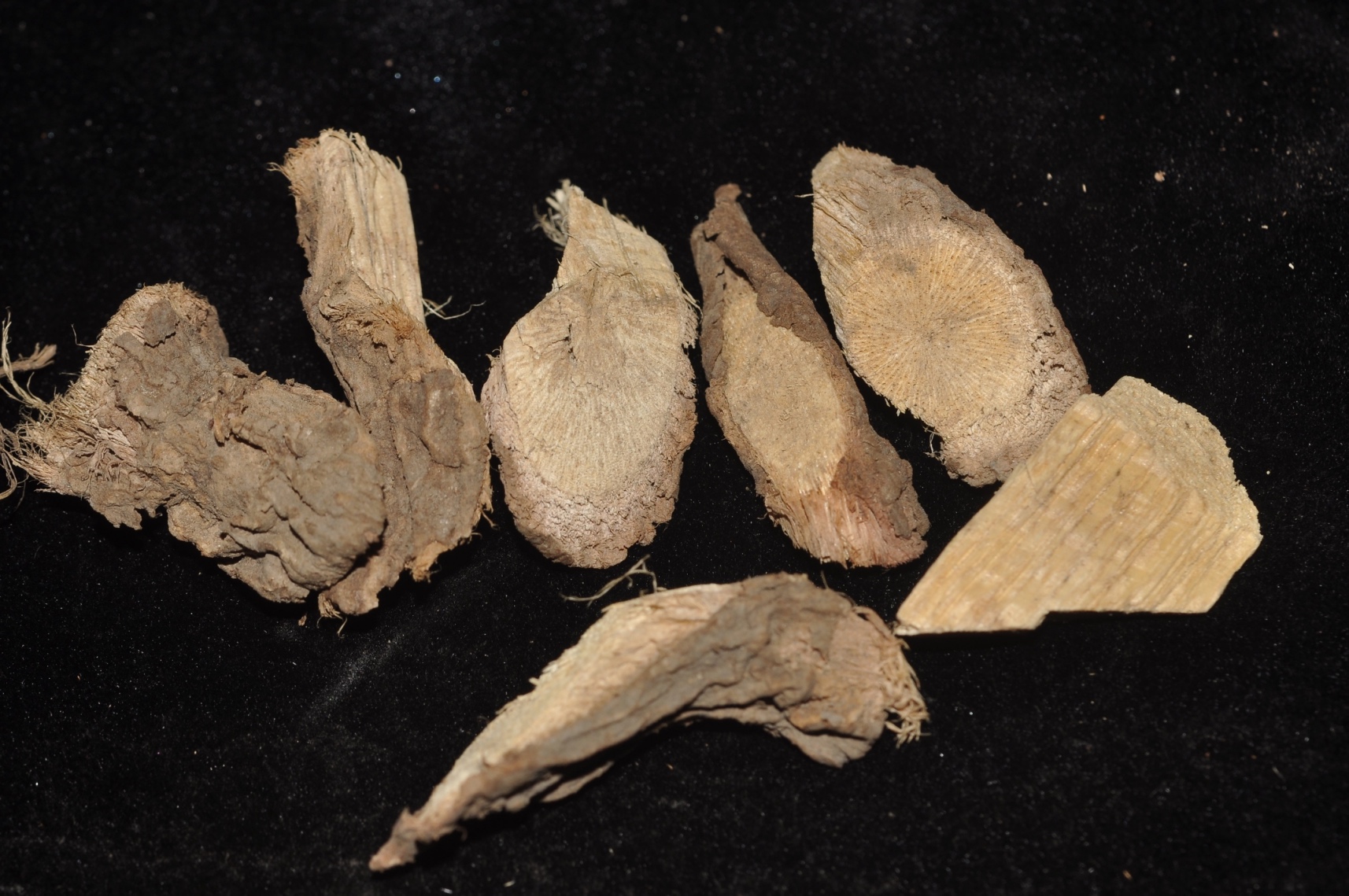

Supplement: Supplementary file 3 [file Data_Sheet_3.zip › Fotor Batch/JPZ07.jpg]

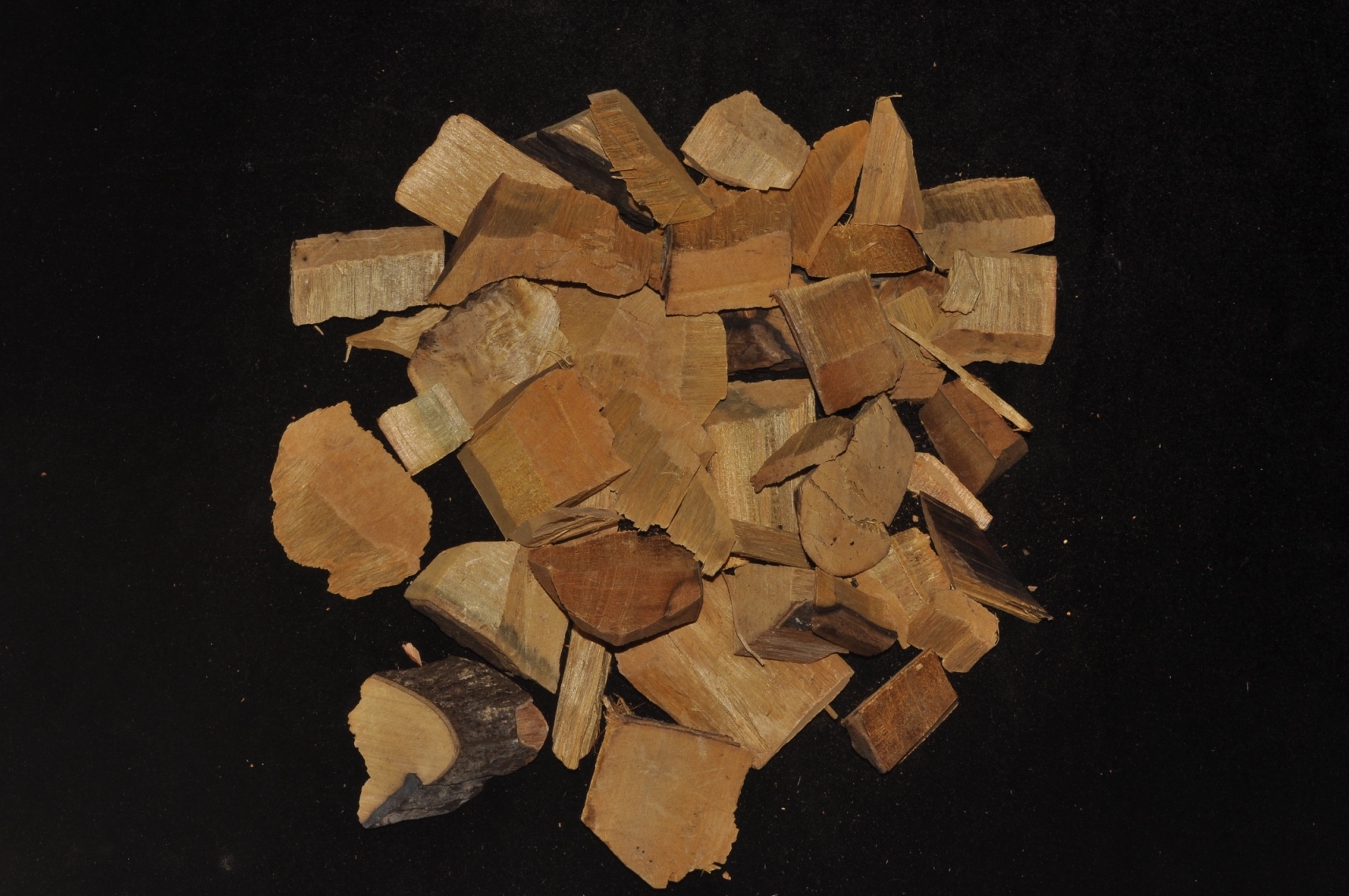

Supplement: Supplementary file 3 [file Data_Sheet_3.zip › Fotor Batch/JPZ06.jpg]

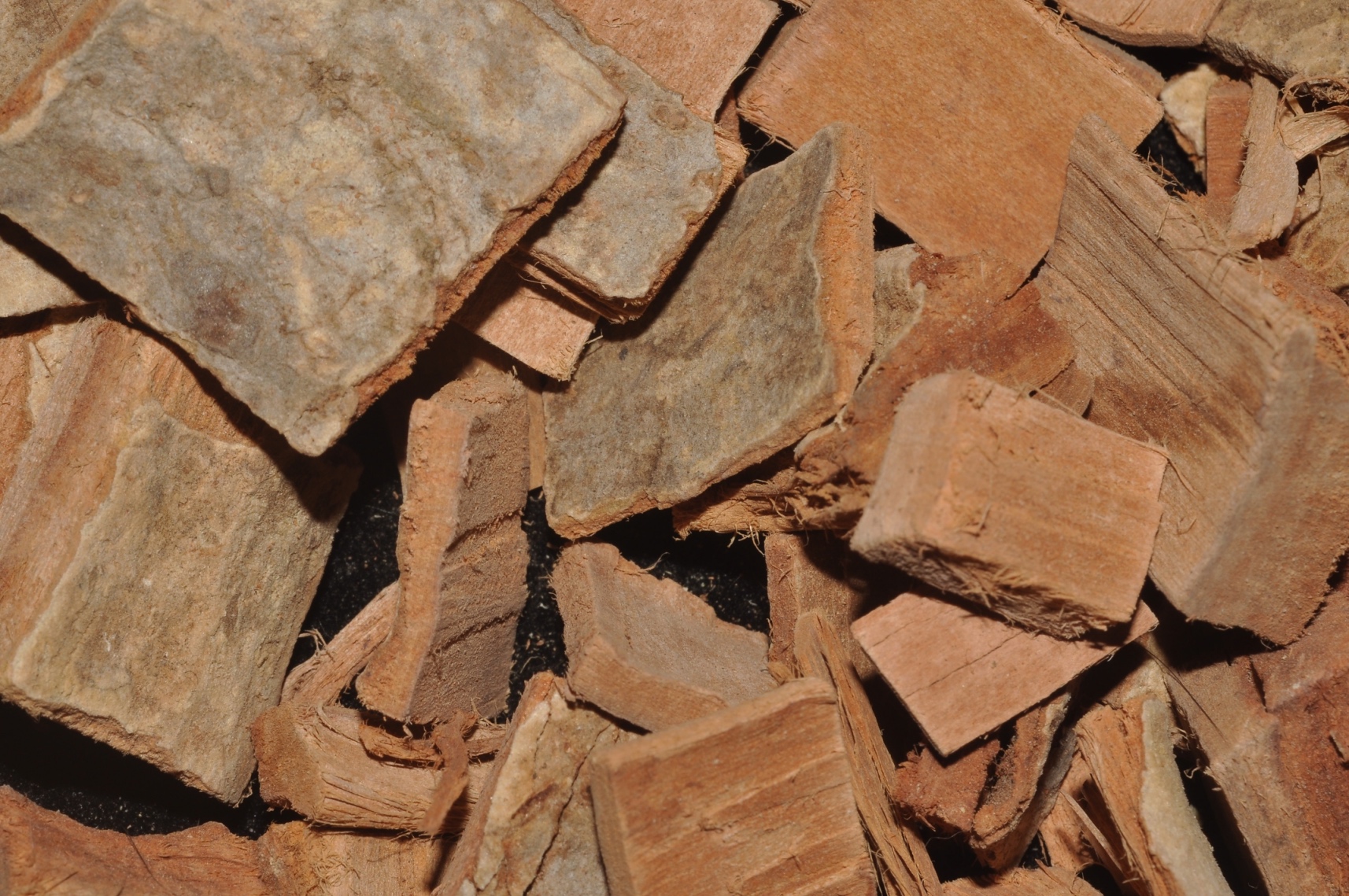

Supplement: Supplementary file 3 [file Data_Sheet_3.zip › Fotor Batch/JPZ12.jpg]

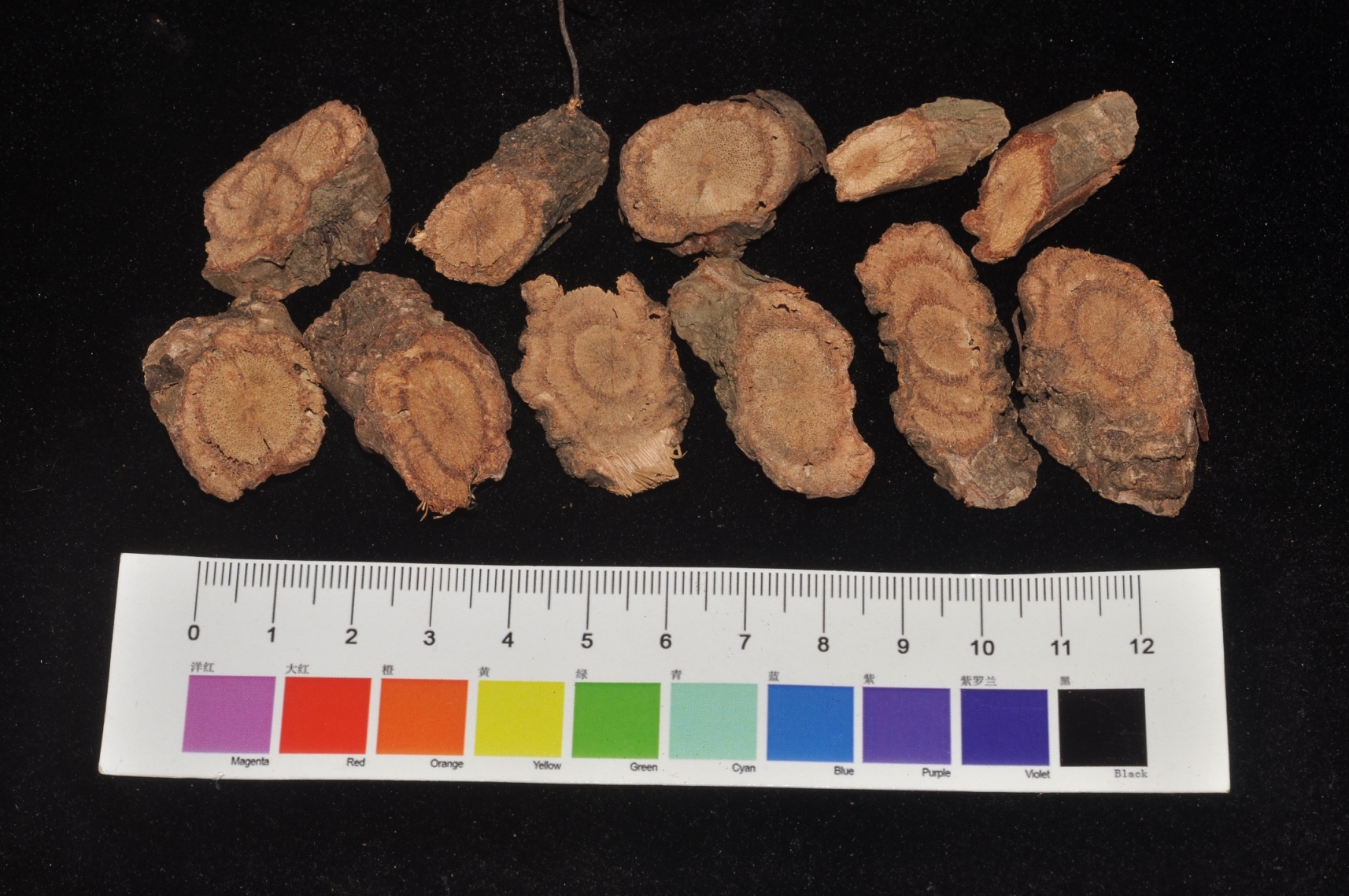

Supplement: Supplementary file 3 [file Data_Sheet_3.zip › Fotor Batch/JPZ37.jpg]

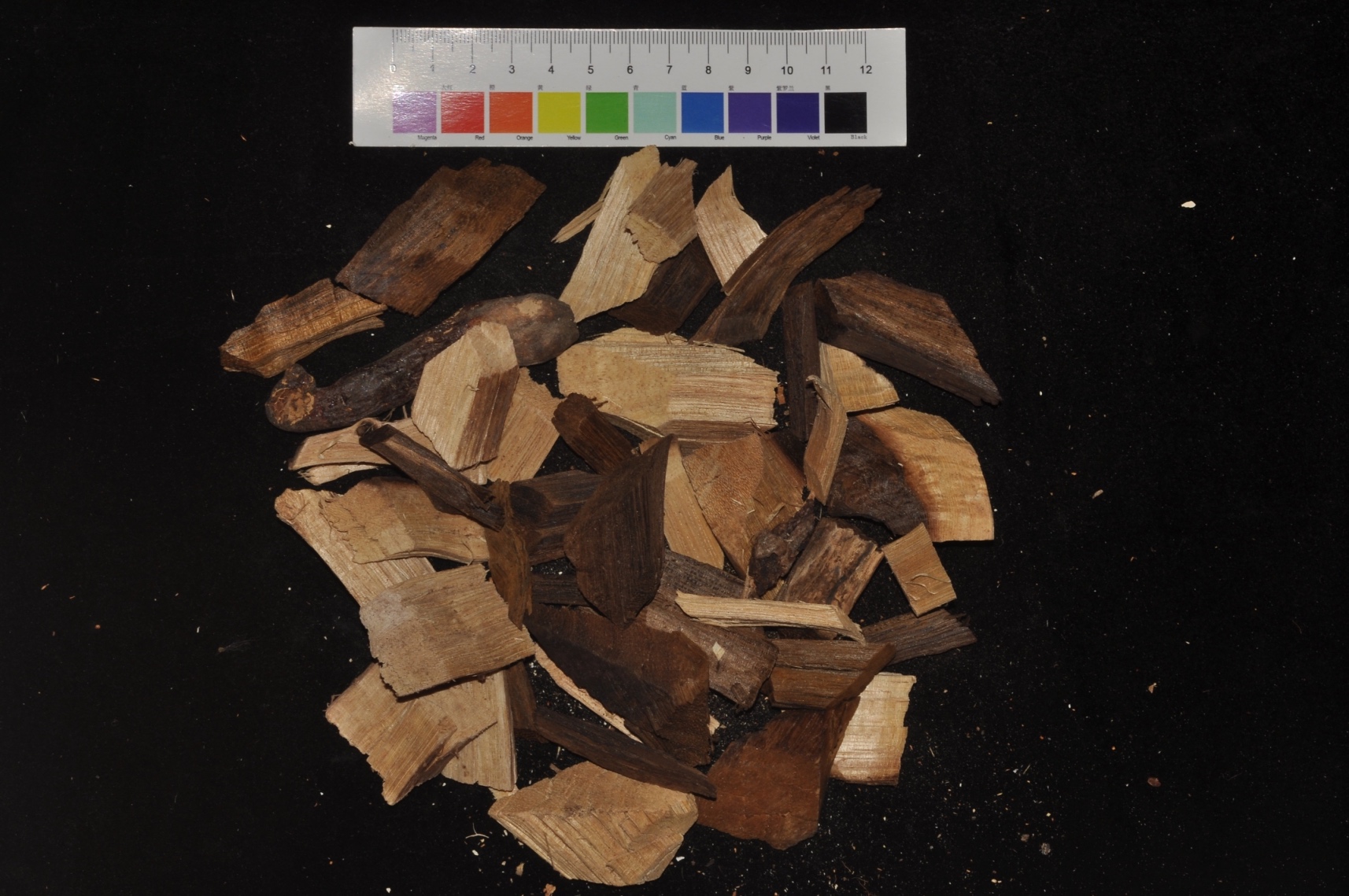

Supplement: Supplementary file 3 [file Data_Sheet_3.zip › Fotor Batch/JPZ23.jpg]

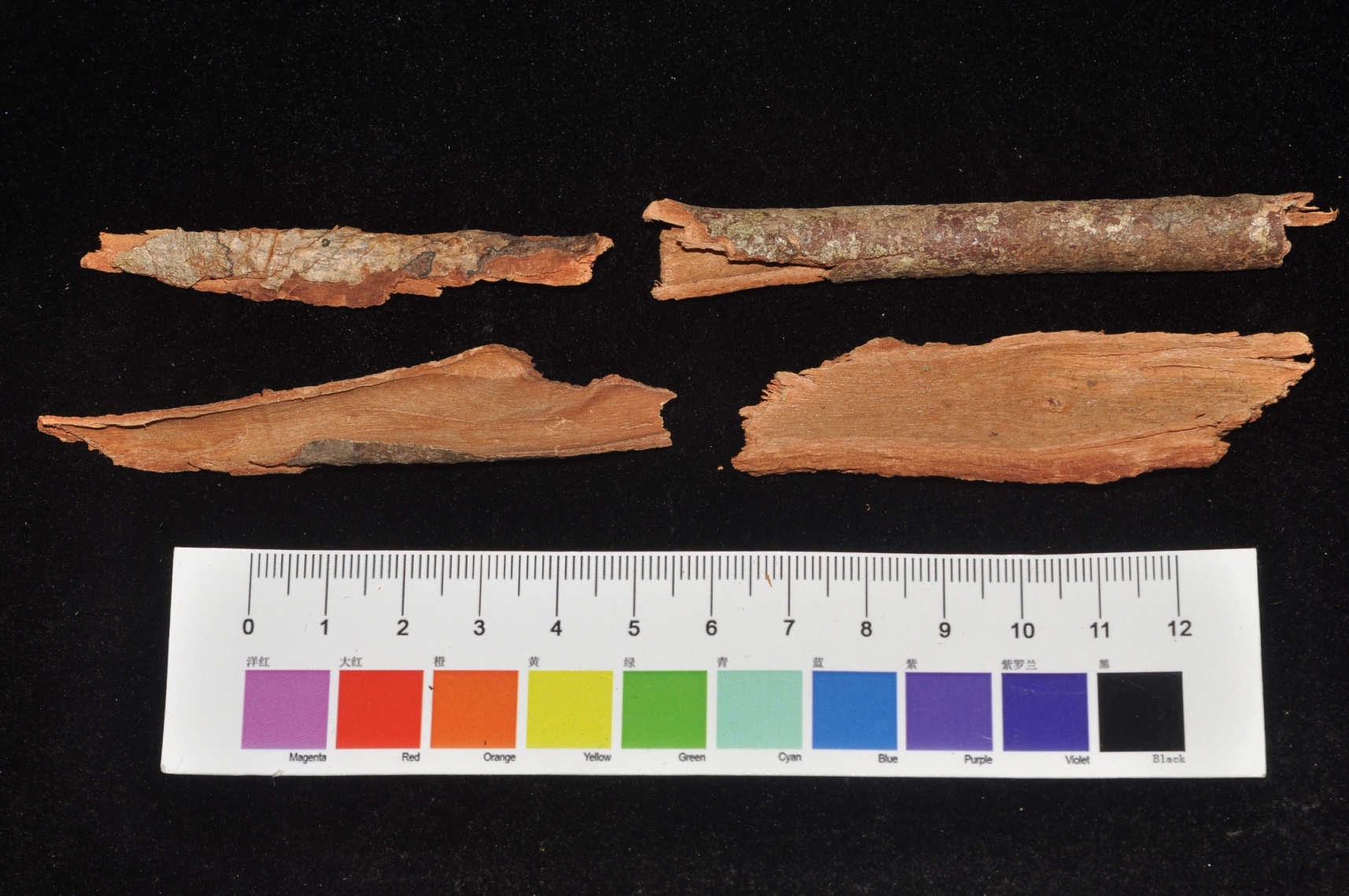

Supplement: Supplementary file 3 [file Data_Sheet_3.zip › Fotor Batch/JPZ22.jpg]

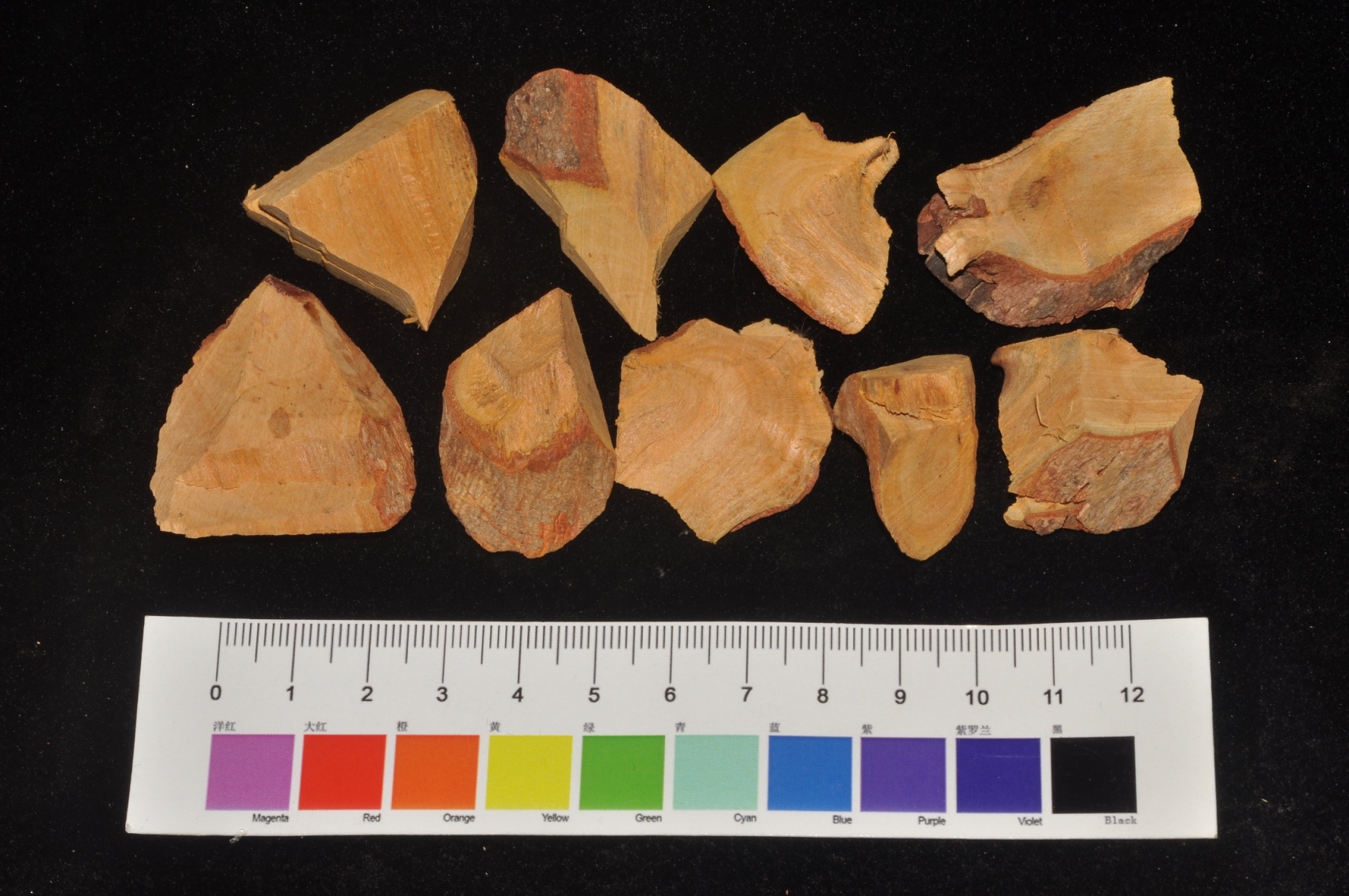

Supplement: Supplementary file 3 [file Data_Sheet_3.zip › Fotor Batch/JPZ36.jpg]

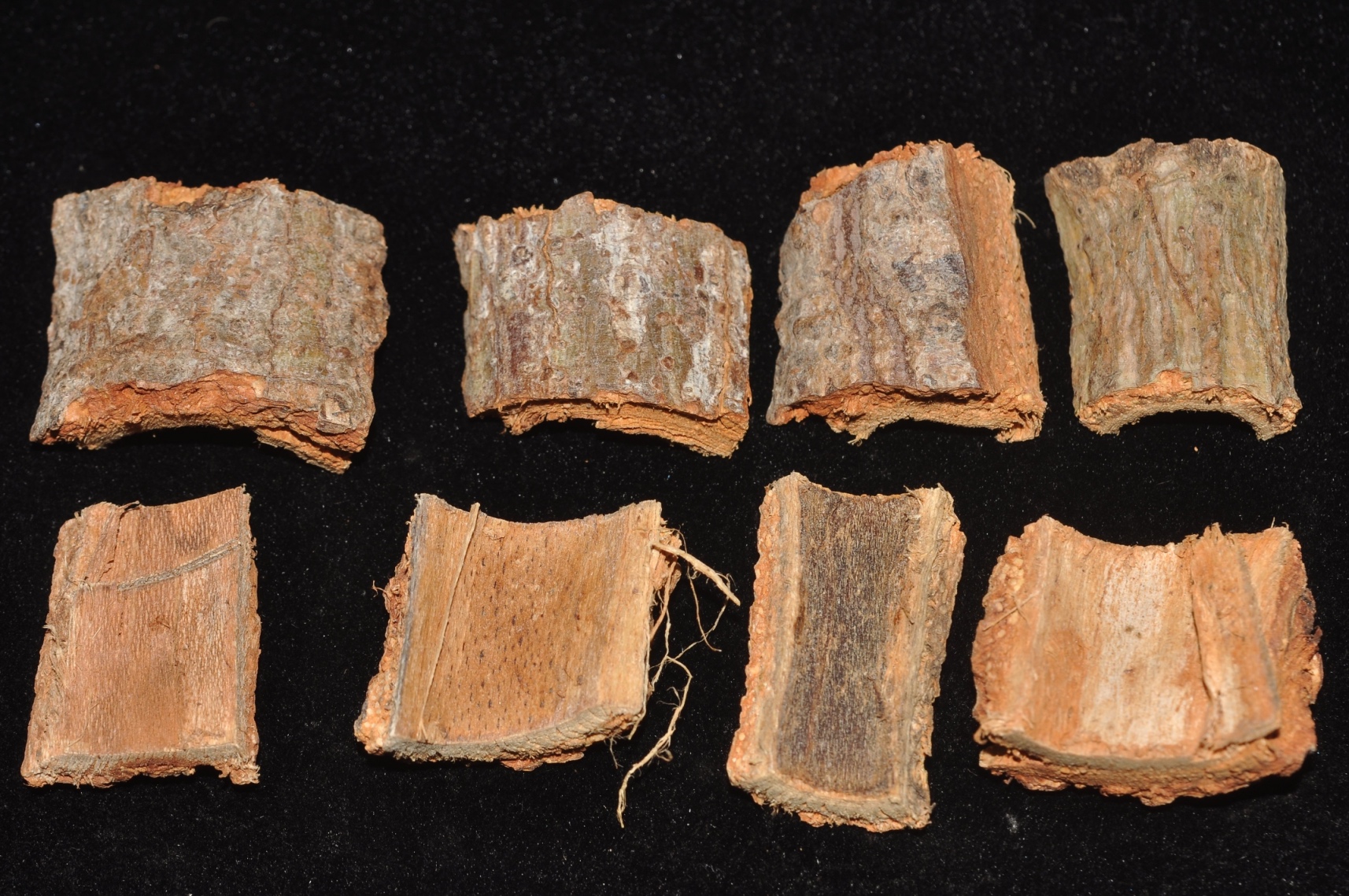

Supplement: Supplementary file 3 [file Data_Sheet_3.zip › Fotor Batch/JPZ08.jpg]

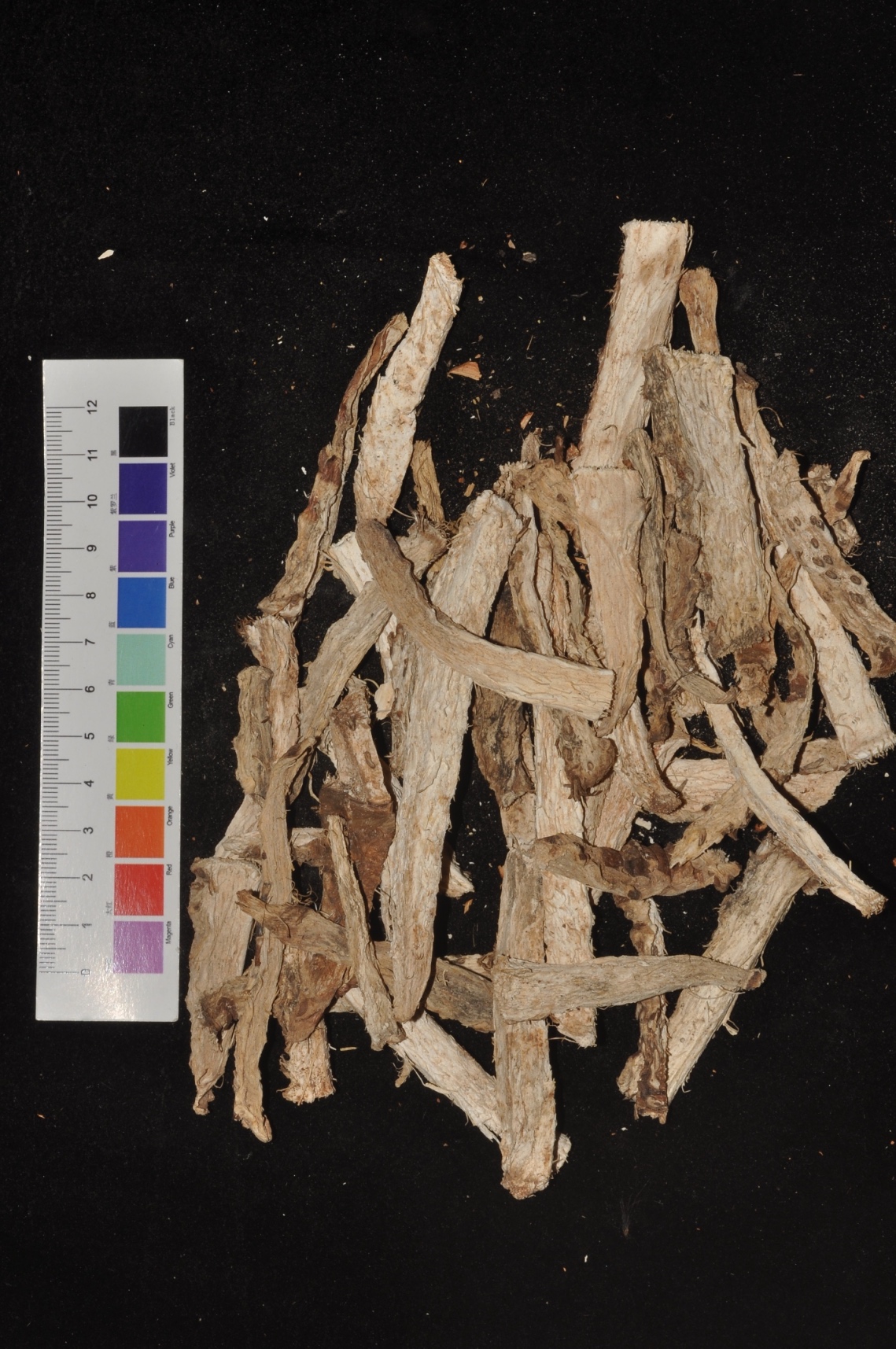

Supplement: Supplementary file 3 [file Data_Sheet_3.zip › Fotor Batch/JPZ20.jpg]

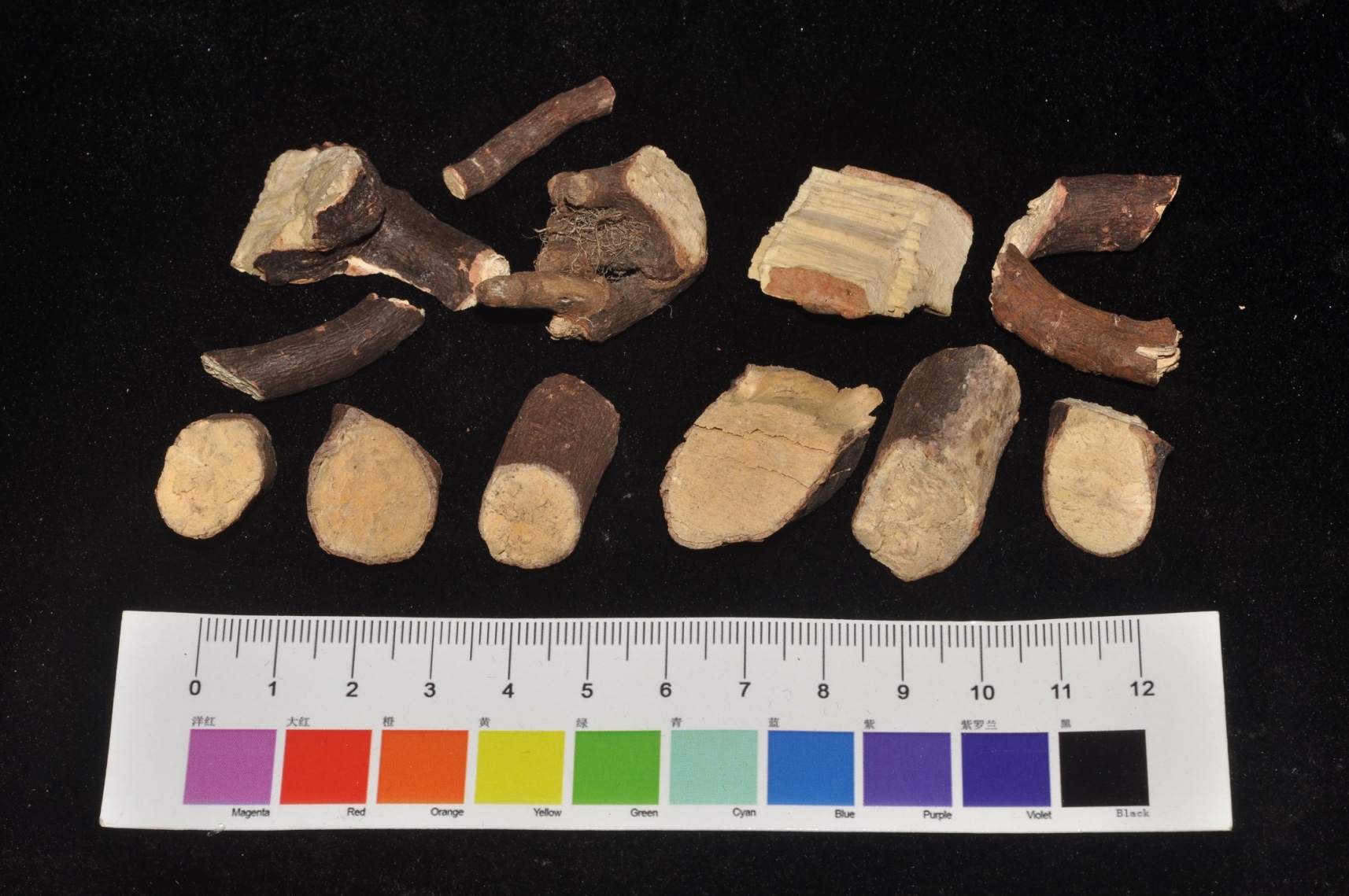

Supplement: Supplementary file 3 [file Data_Sheet_3.zip › Fotor Batch/JPZ34.jpg]

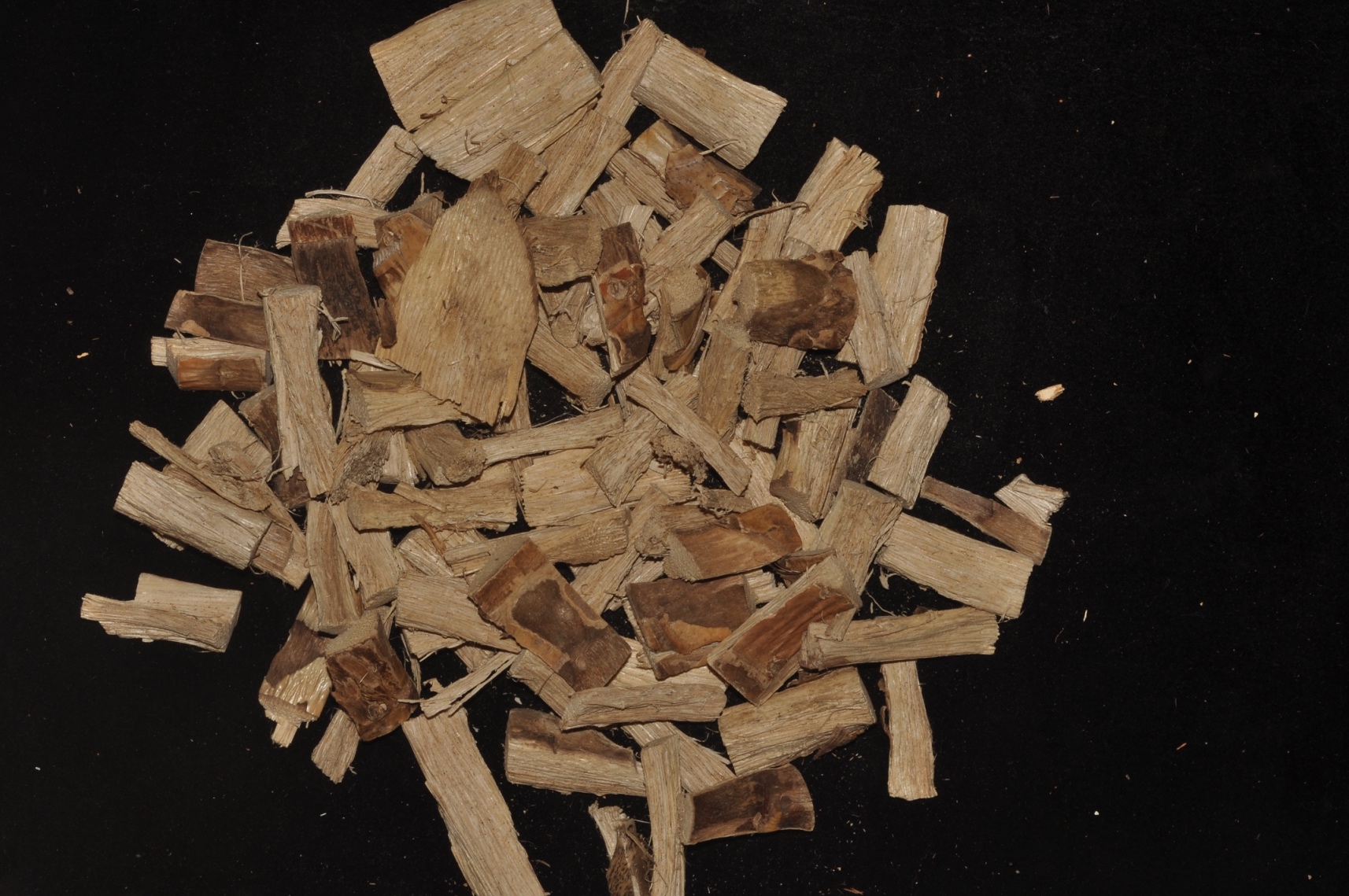

Supplement: Supplementary file 3 [file Data_Sheet_3.zip › Fotor Batch/JPZ35.jpg]

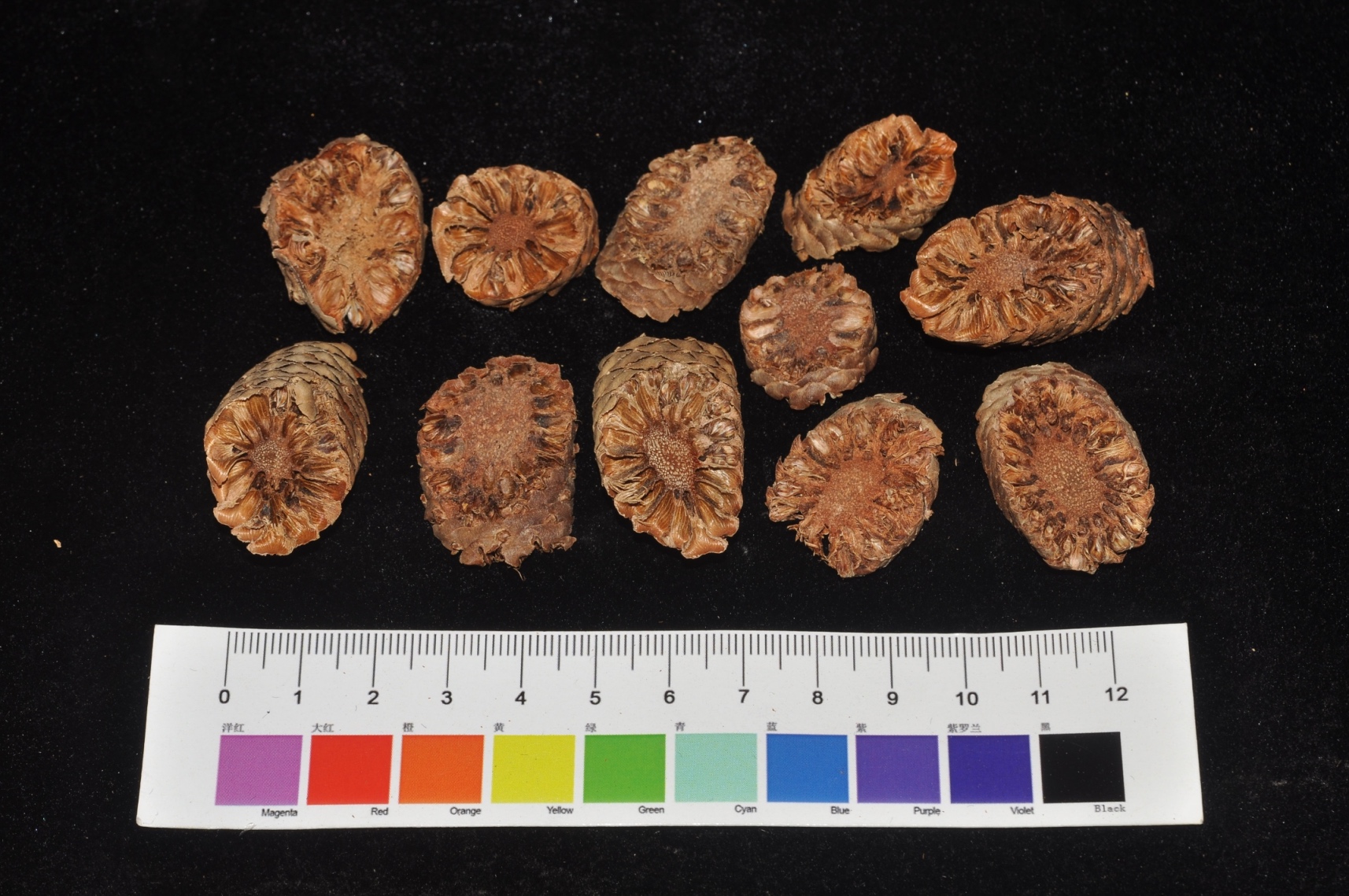

Supplement: Supplementary file 3 [file Data_Sheet_3.zip › Fotor Batch/JPZ21.jpg]

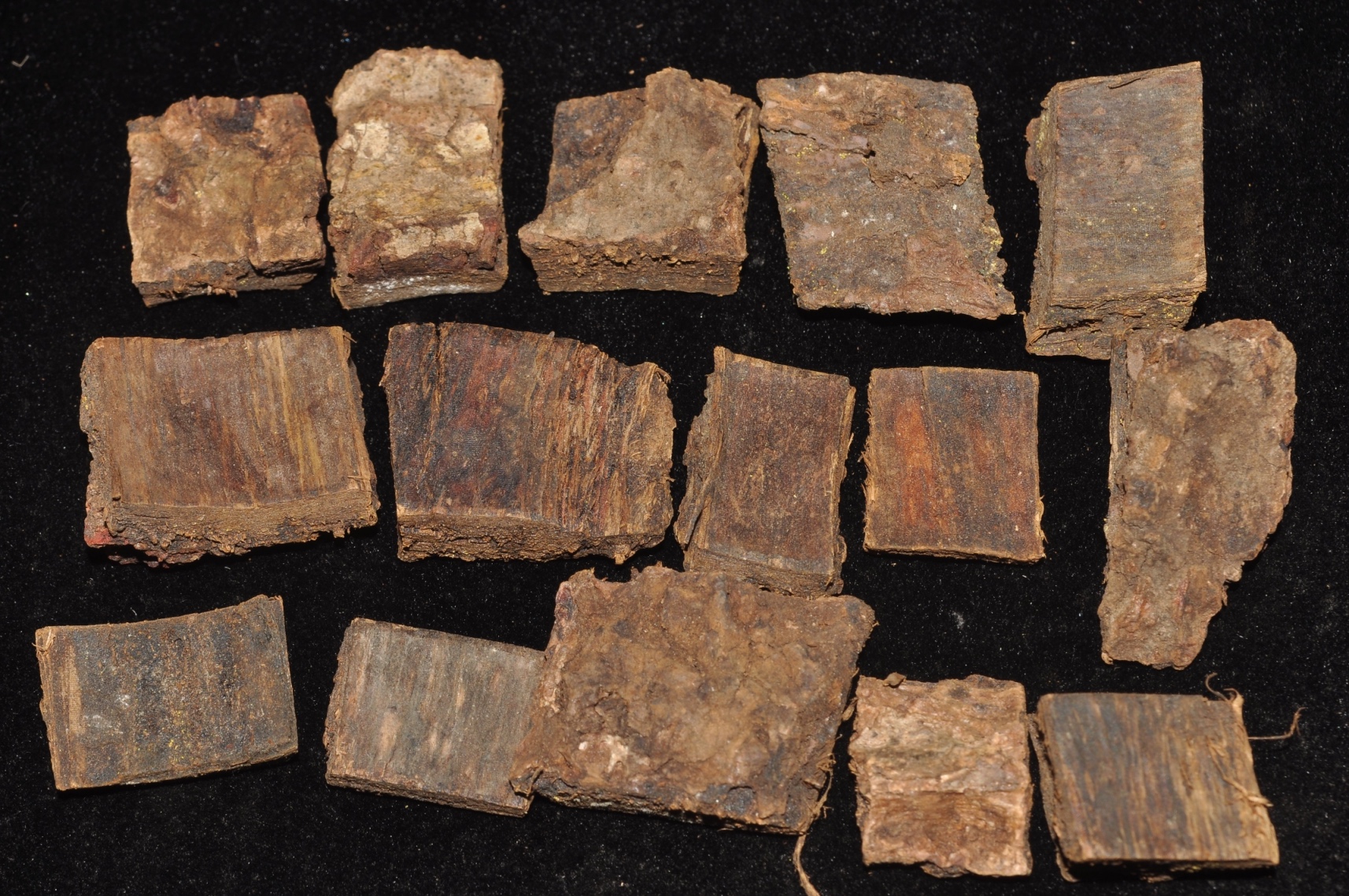

Supplement: Supplementary file 3 [file Data_Sheet_3.zip › Fotor Batch/JPZ09.jpg]

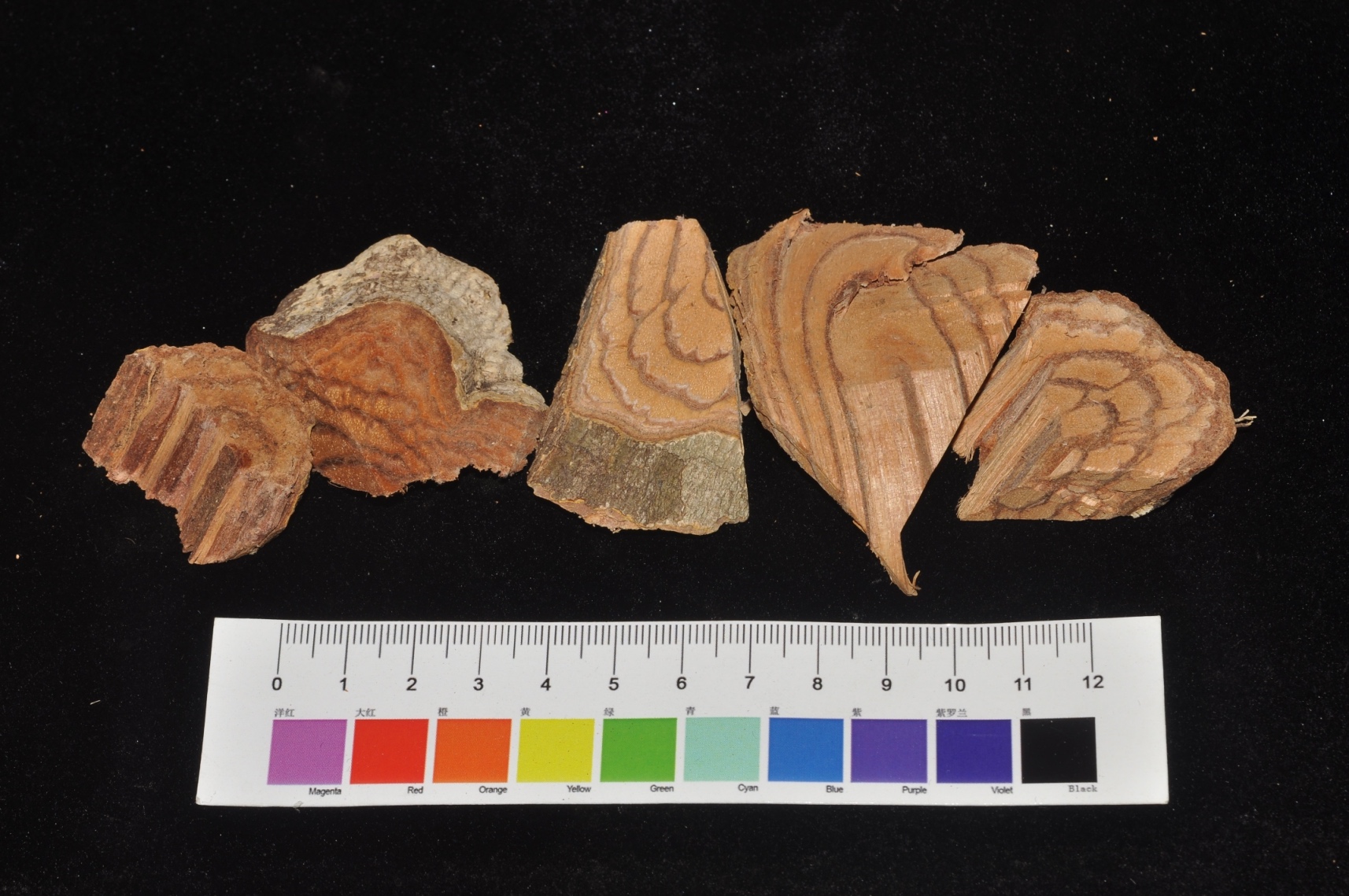

Supplement: Supplementary file 3 [file Data_Sheet_3.zip › Fotor Batch/JPZ25.jpg]

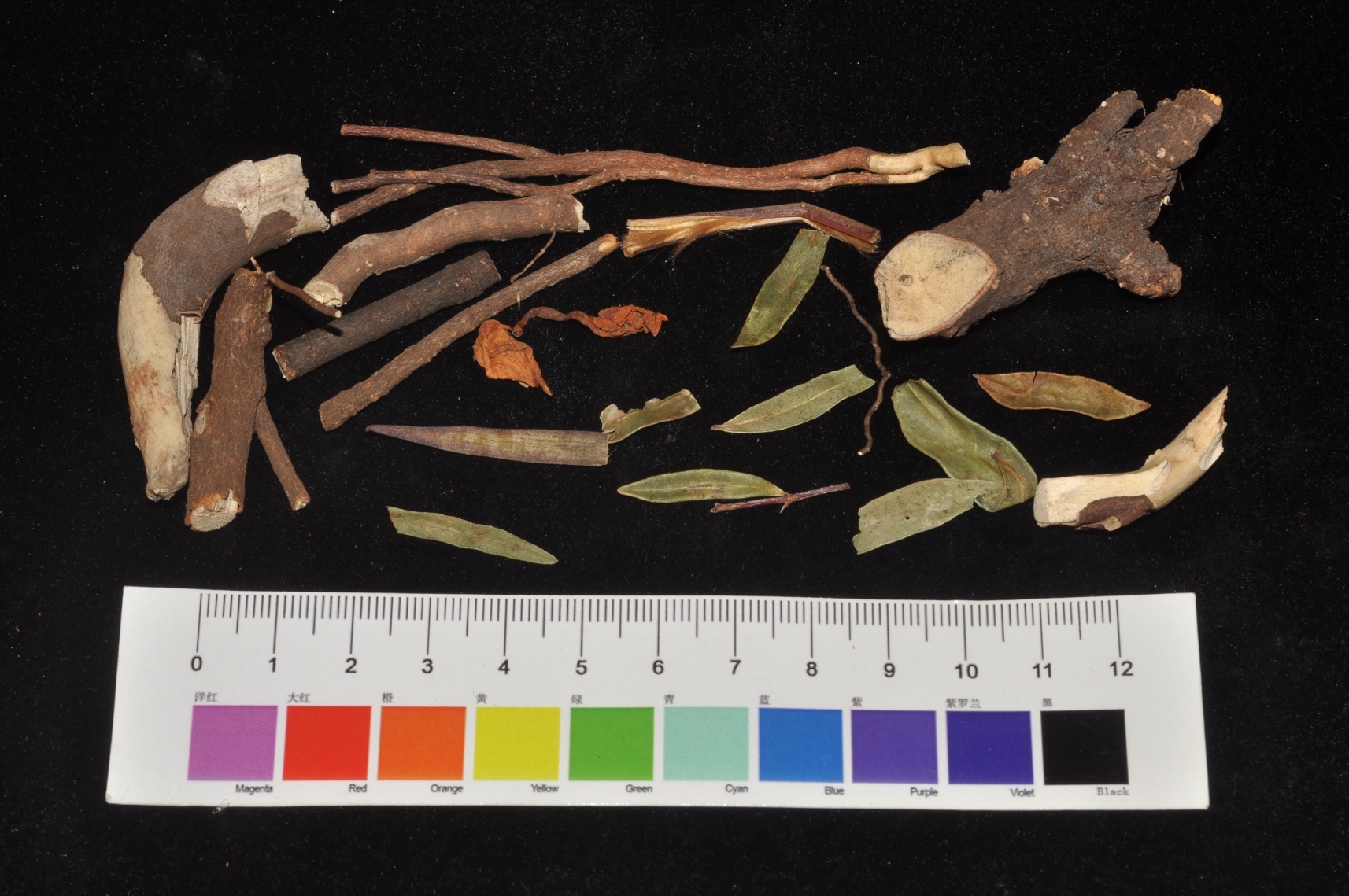

Supplement: Supplementary file 3 [file Data_Sheet_3.zip › Fotor Batch/JPZ31.jpg]

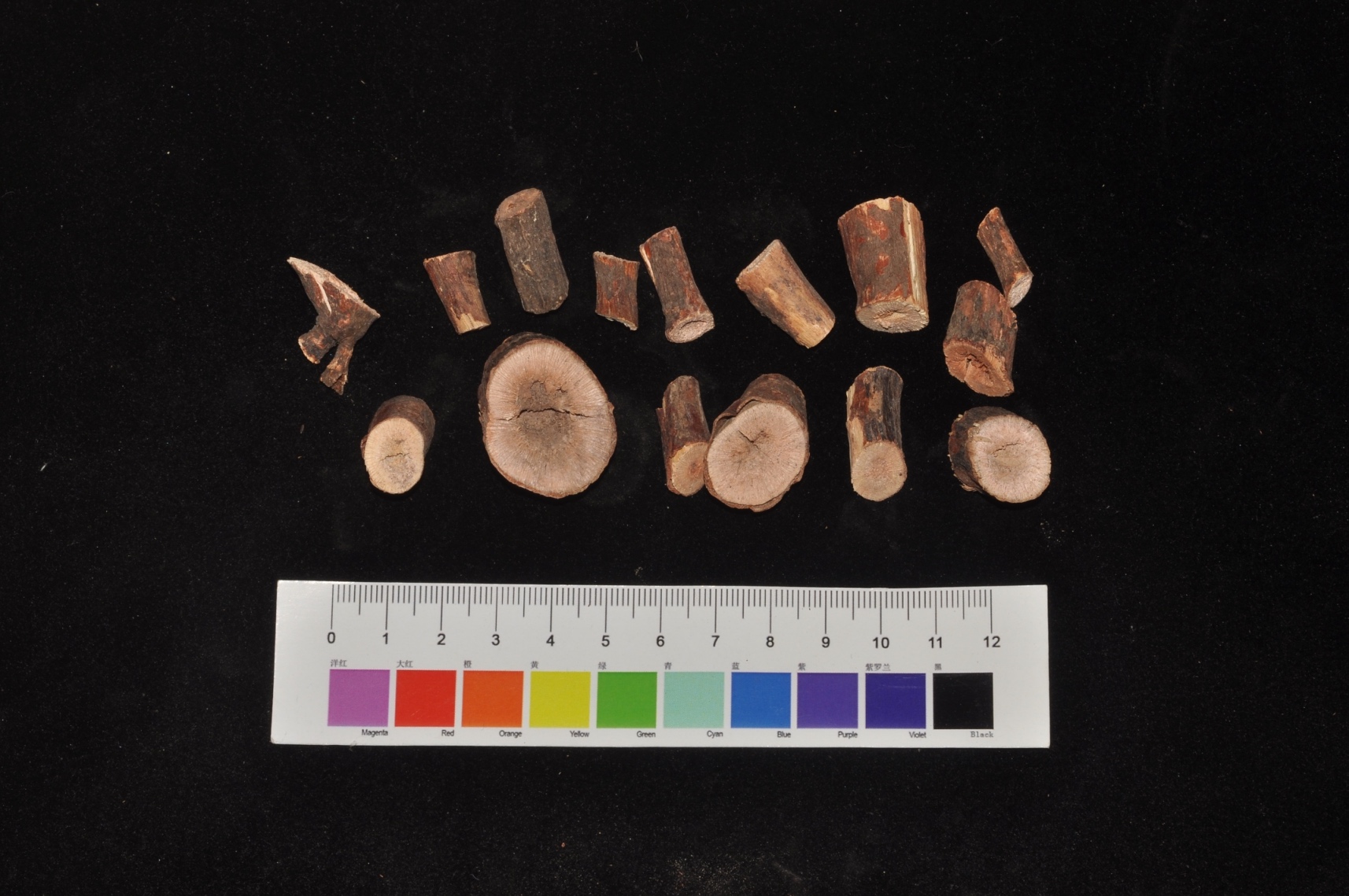

Supplement: Supplementary file 3 [file Data_Sheet_3.zip › Fotor Batch/JPZ19.jpg]

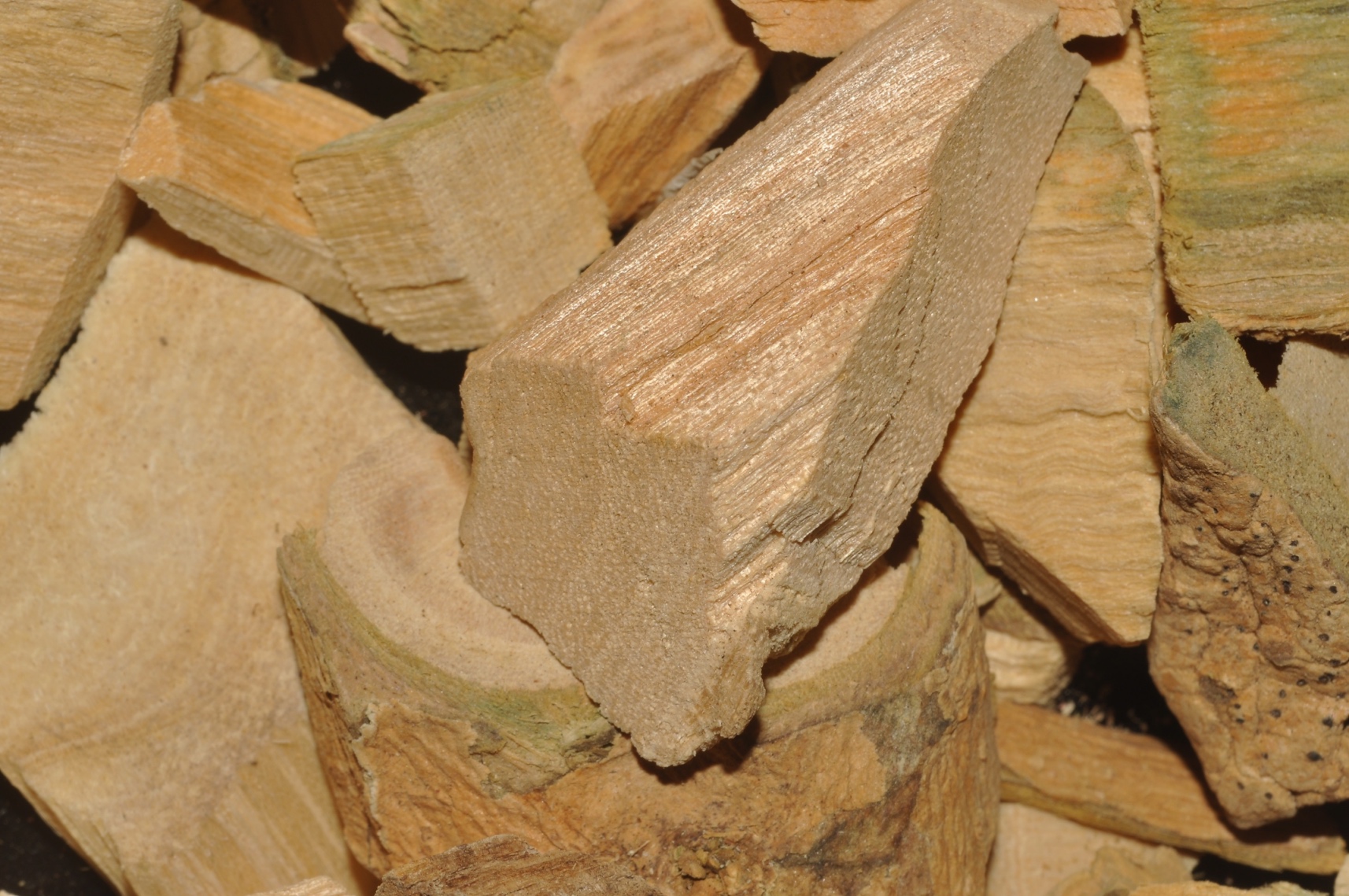

Supplement: Supplementary file 3 [file Data_Sheet_3.zip › Fotor Batch/JPZ18.jpg]

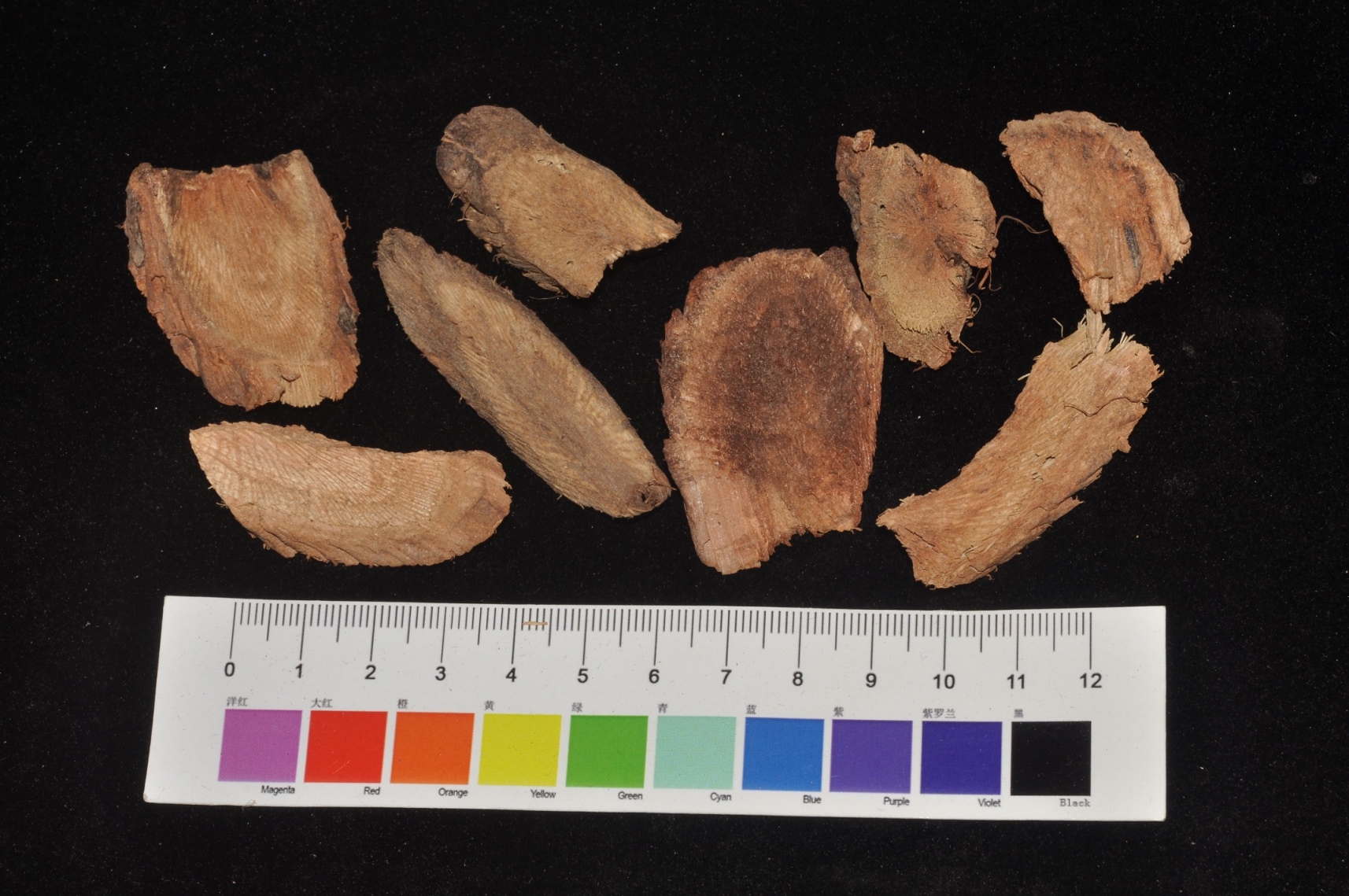

Supplement: Supplementary file 3 [file Data_Sheet_3.zip › Fotor Batch/JPZ30.jpg]

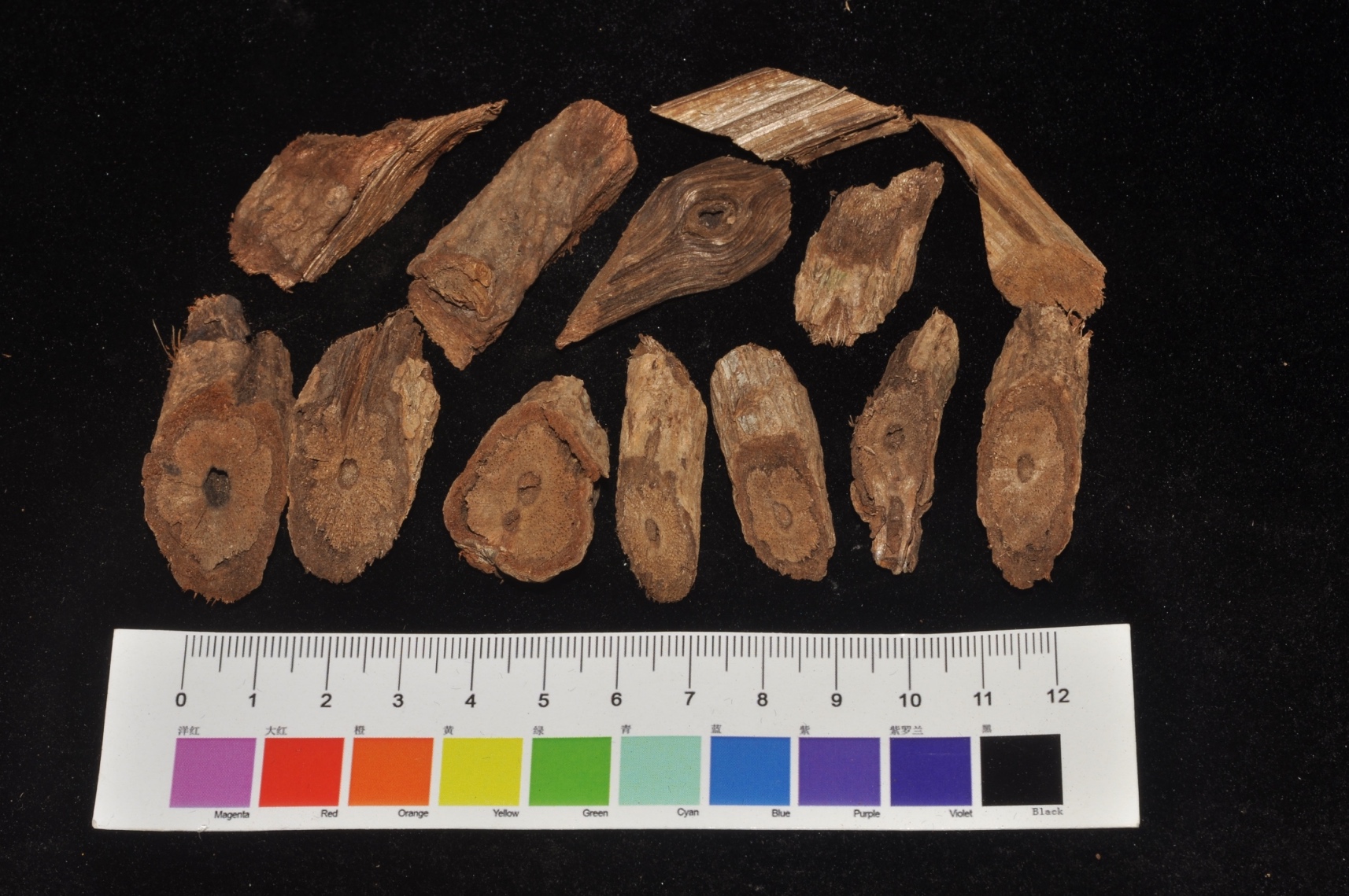

Supplement: Supplementary file 3 [file Data_Sheet_3.zip › Fotor Batch/JPZ24.jpg]

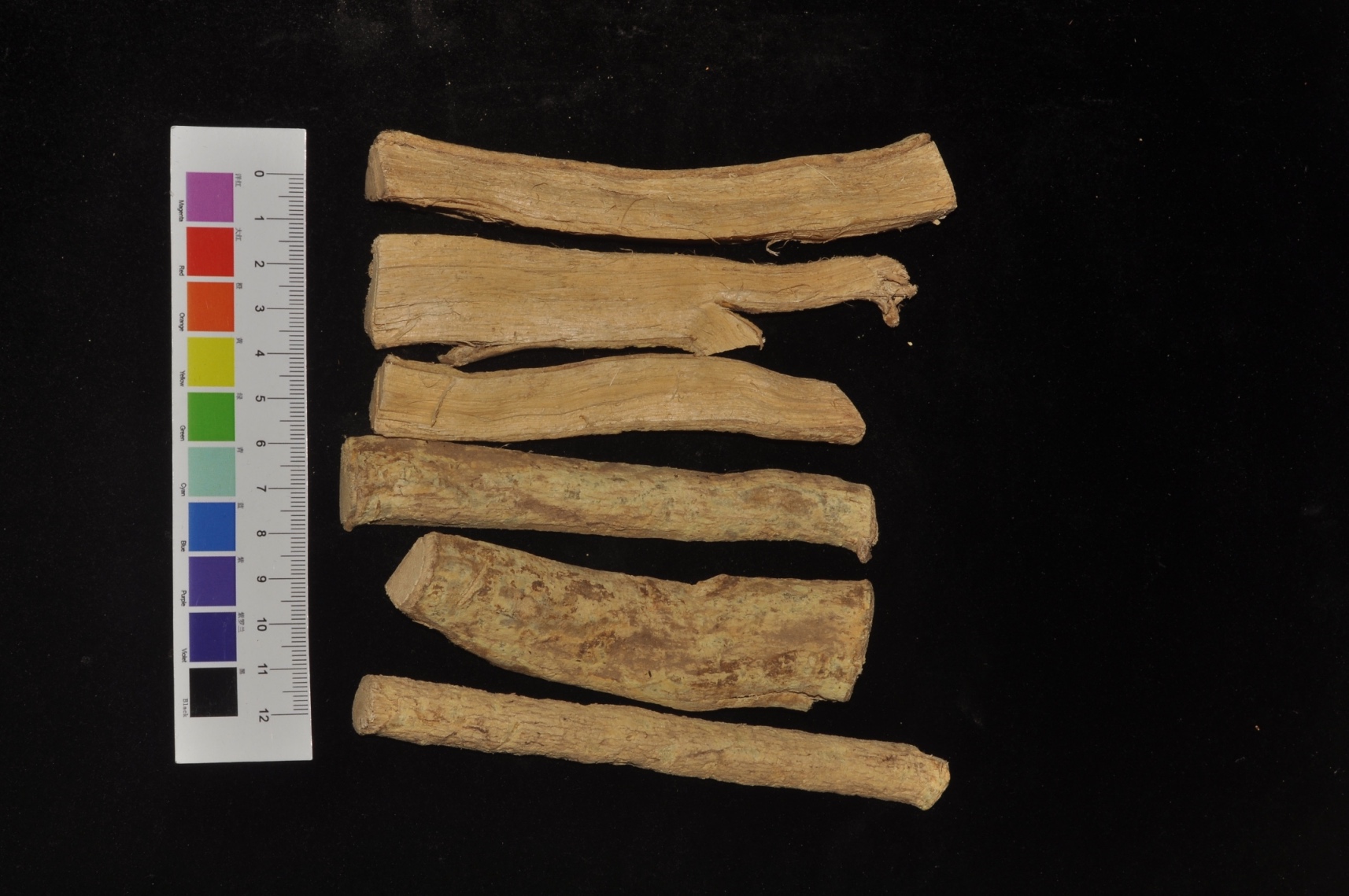

Supplement: Supplementary file 3 [file Data_Sheet_3.zip › Fotor Batch/JPZ32.jpg]

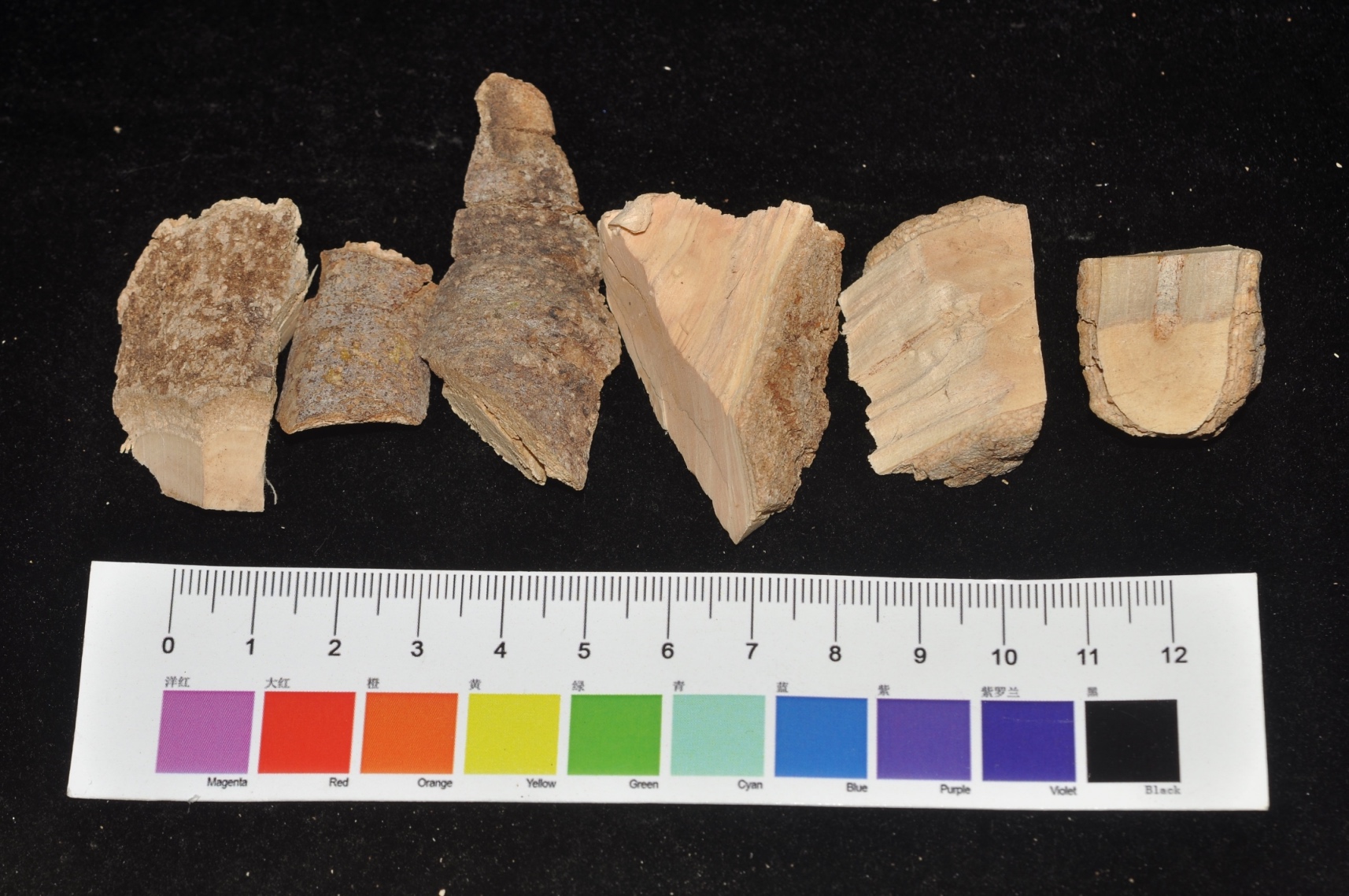

Supplement: Supplementary file 3 [file Data_Sheet_3.zip › Fotor Batch/JPZ26.jpg]

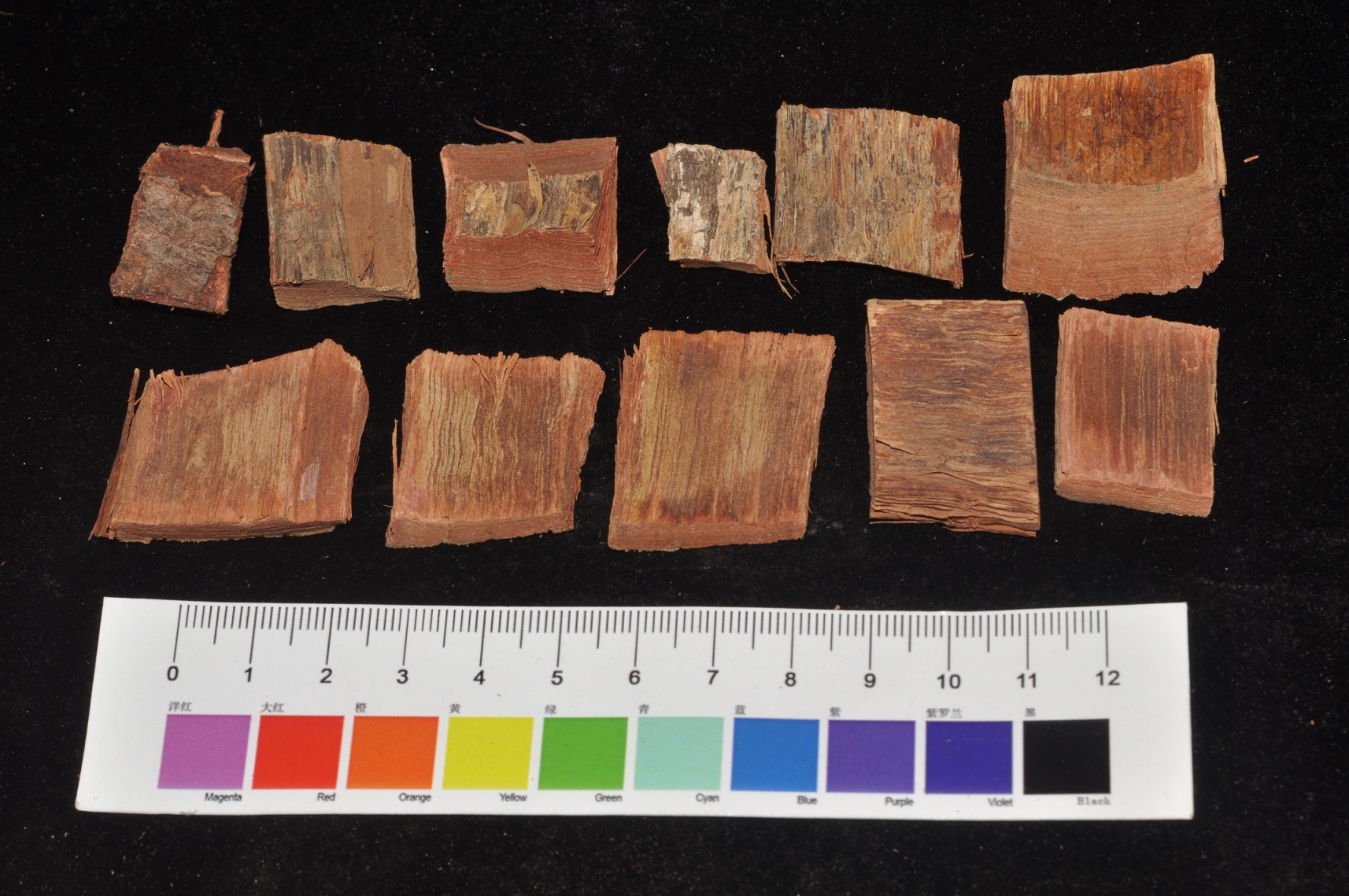

Supplement: Supplementary file 3 [file Data_Sheet_3.zip › Fotor Batch/JPZ27.jpg]

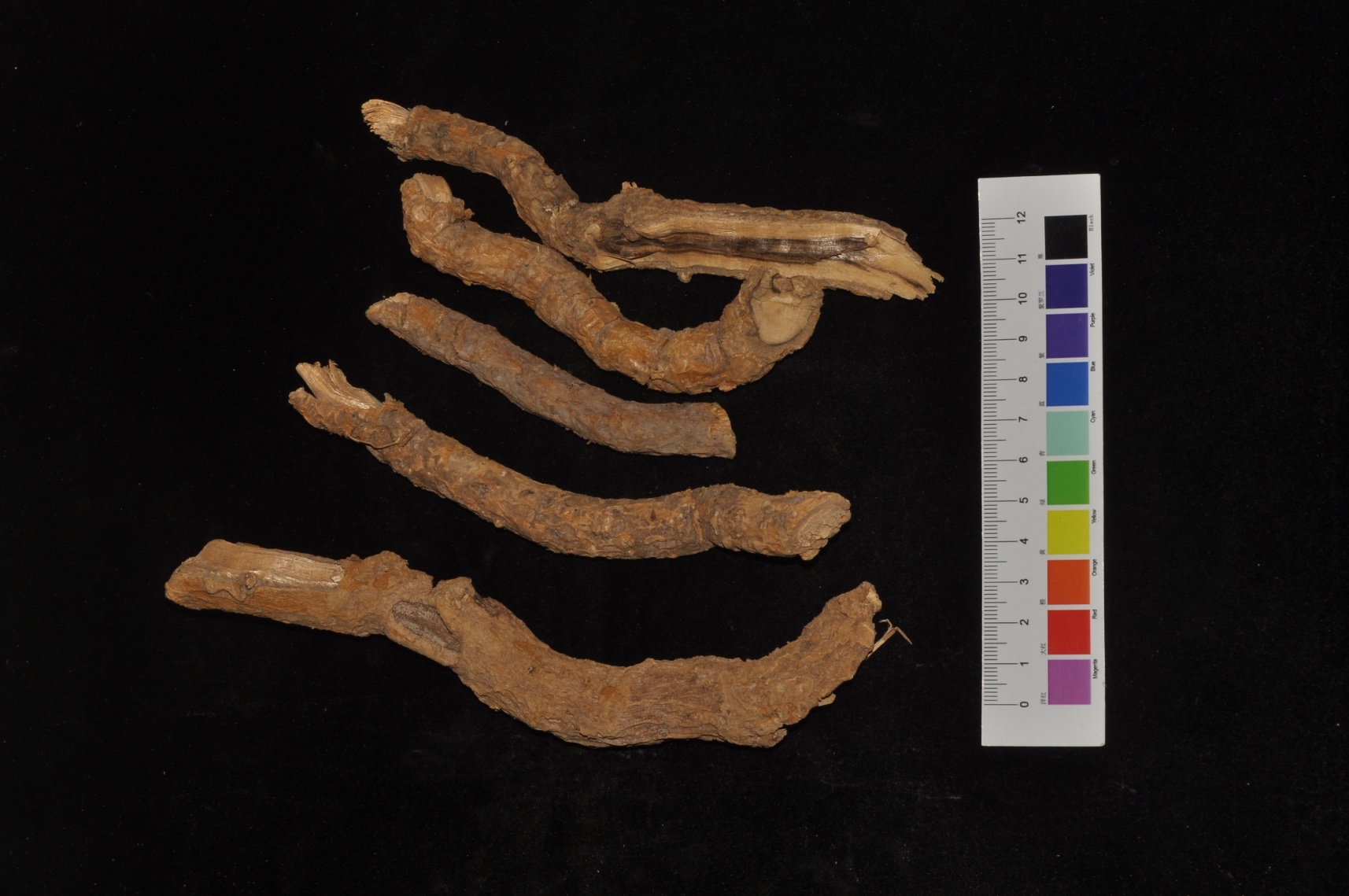

Supplement: Supplementary file 3 [file Data_Sheet_3.zip › Fotor Batch/JPZ33.jpg]

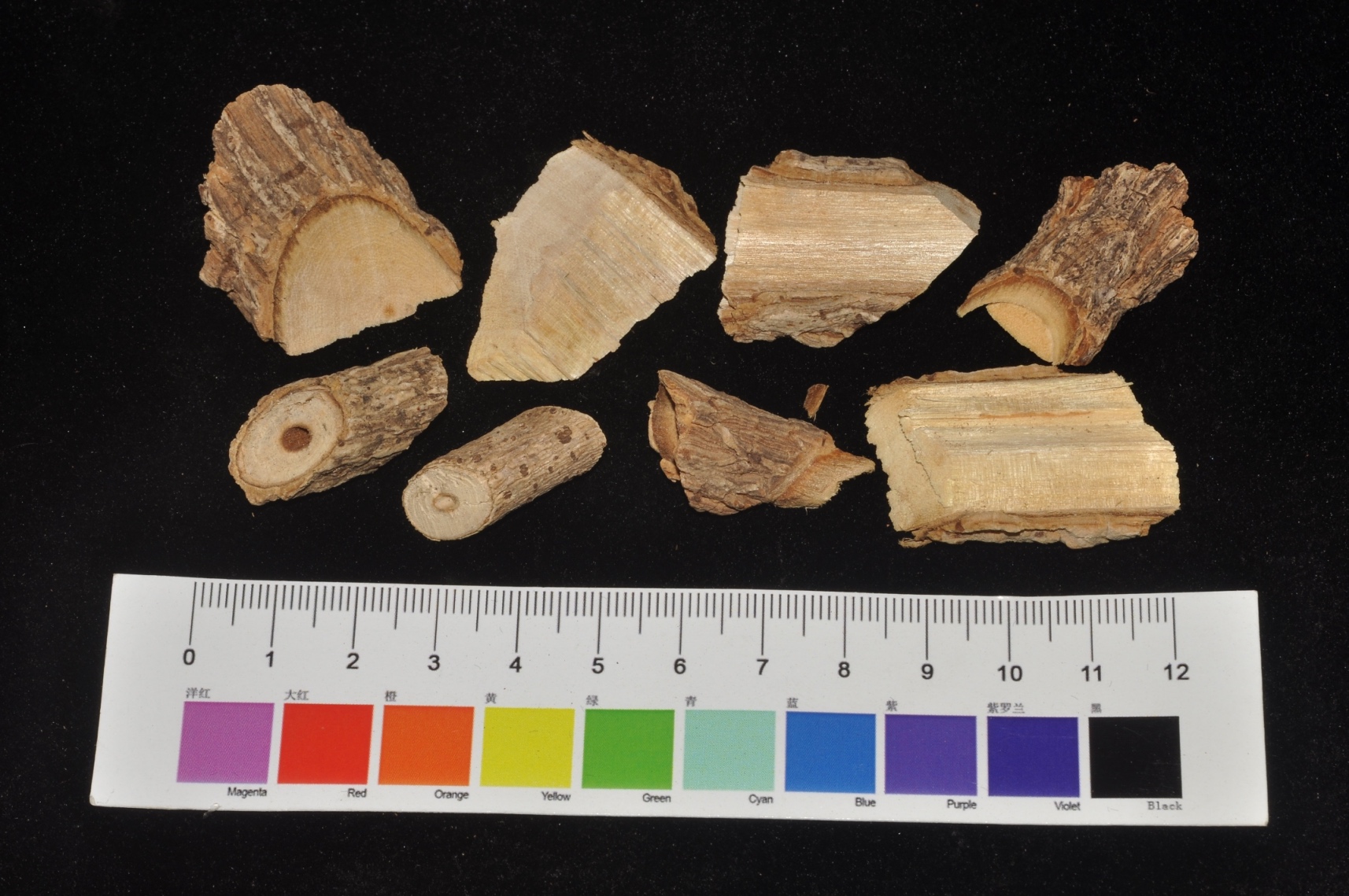

Supplement: Supplementary file 3 [file Data_Sheet_3.zip › Fotor Batch/JPZ40.jpg]

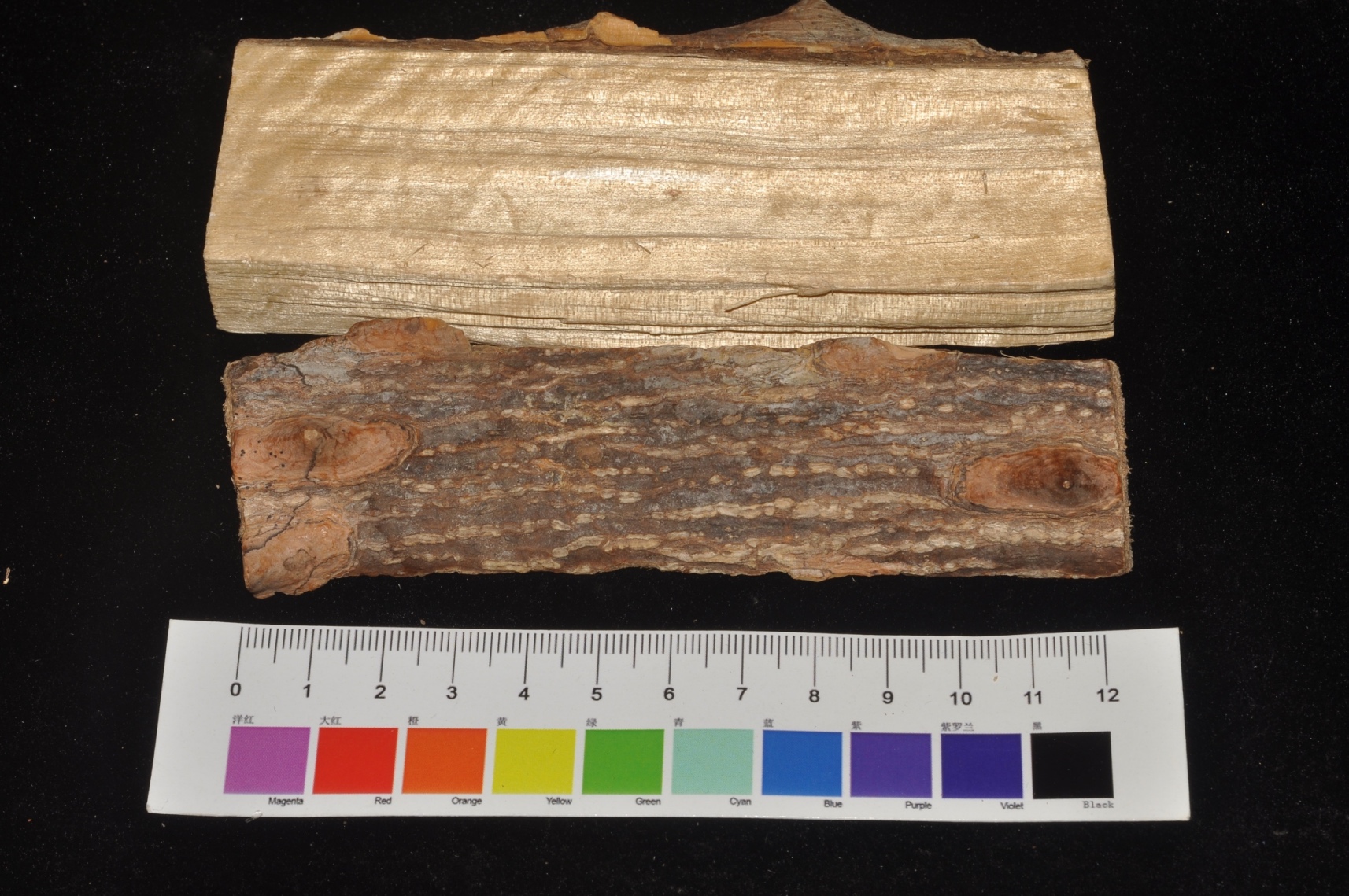

Supplement: Supplementary file 3 [file Data_Sheet_3.zip › Fotor Batch/JPZ41.jpg]

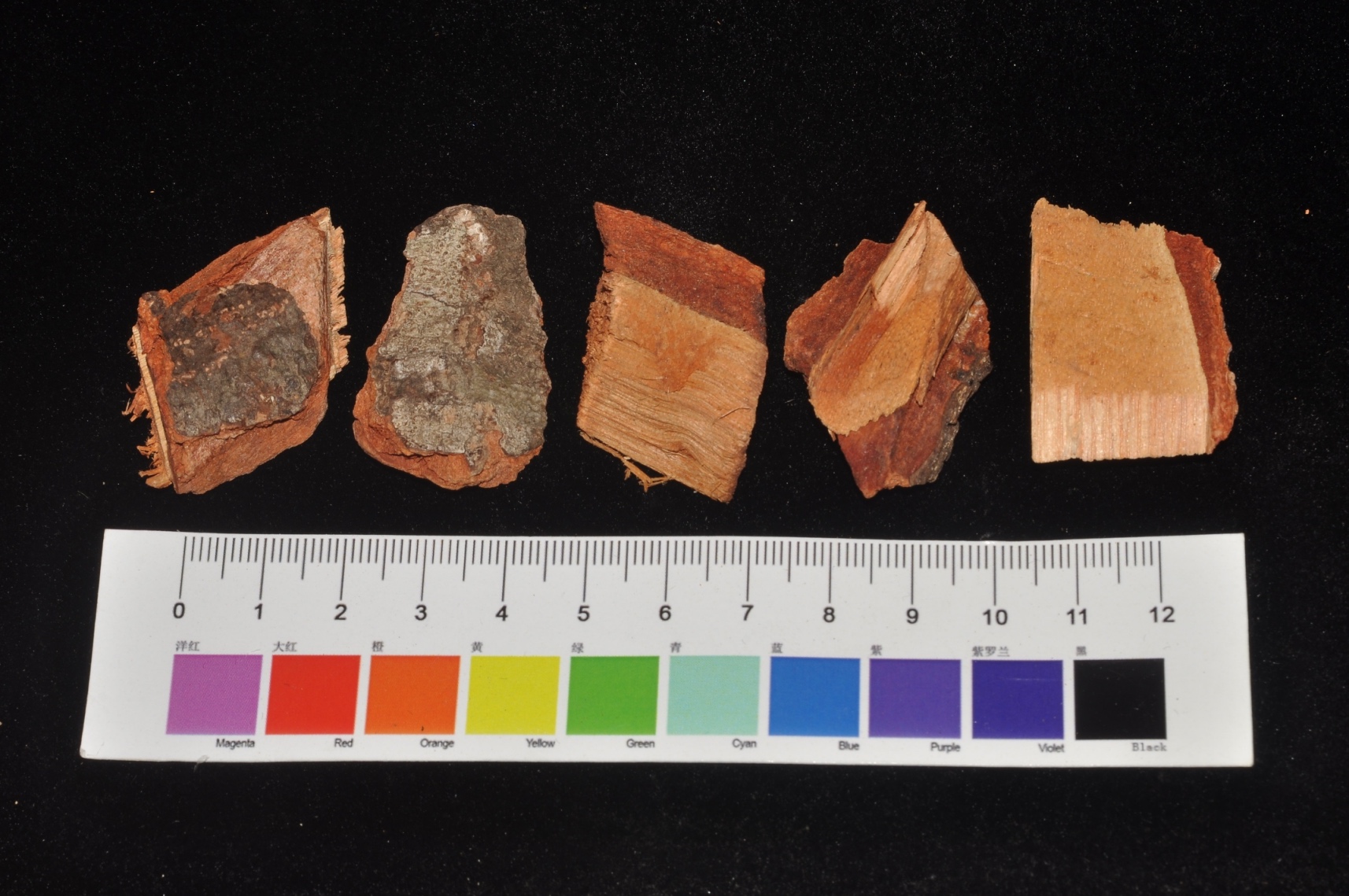

Supplement: Supplementary file 3 [file Data_Sheet_3.zip › Fotor Batch/JPZ43.jpg]

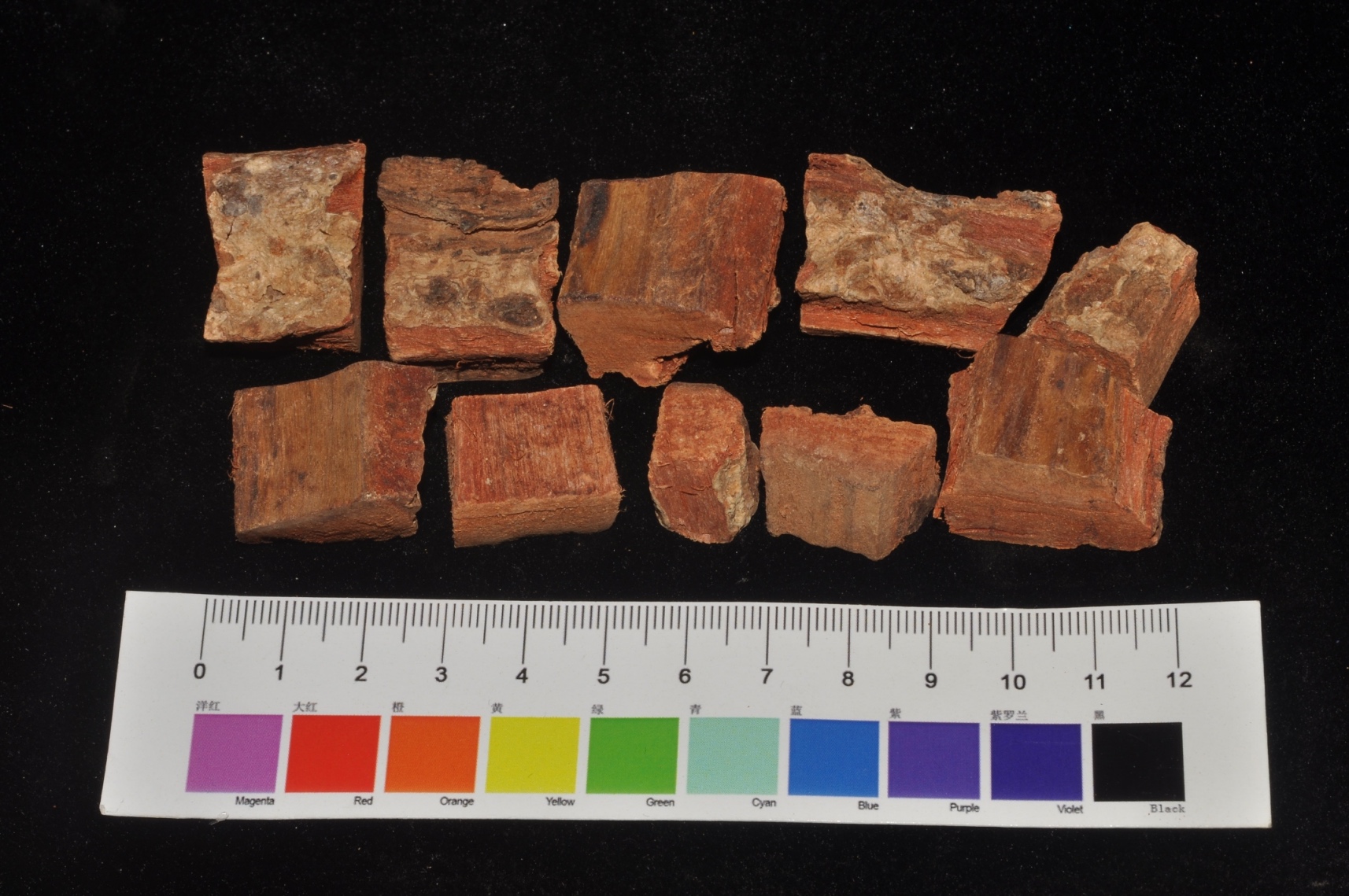

Supplement: Supplementary file 3 [file Data_Sheet_3.zip › Fotor Batch/JPZ42.jpg]

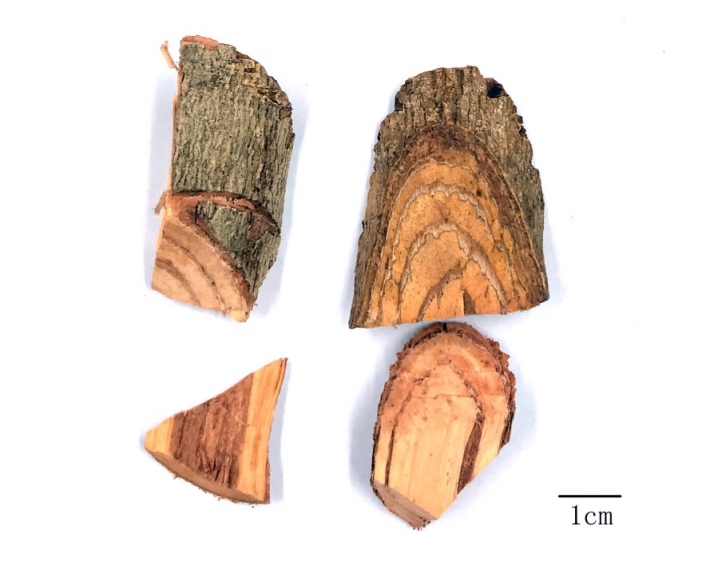

Supplement: Supplementary file 3 [file Data_Sheet_3.zip › Fotor Batch/JPZ46.jpg]

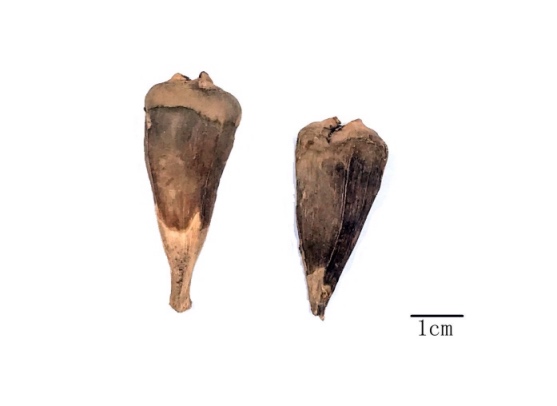

Supplement: Supplementary file 3 [file Data_Sheet_3.zip › Fotor Batch/JPZ47.jpg]

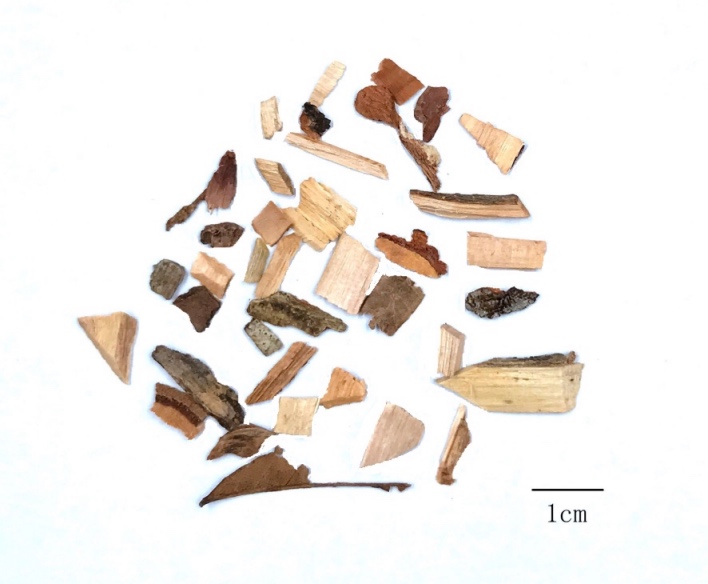

Supplement: Supplementary file 3 [file Data_Sheet_3.zip › Fotor Batch/JPZ45.jpg]

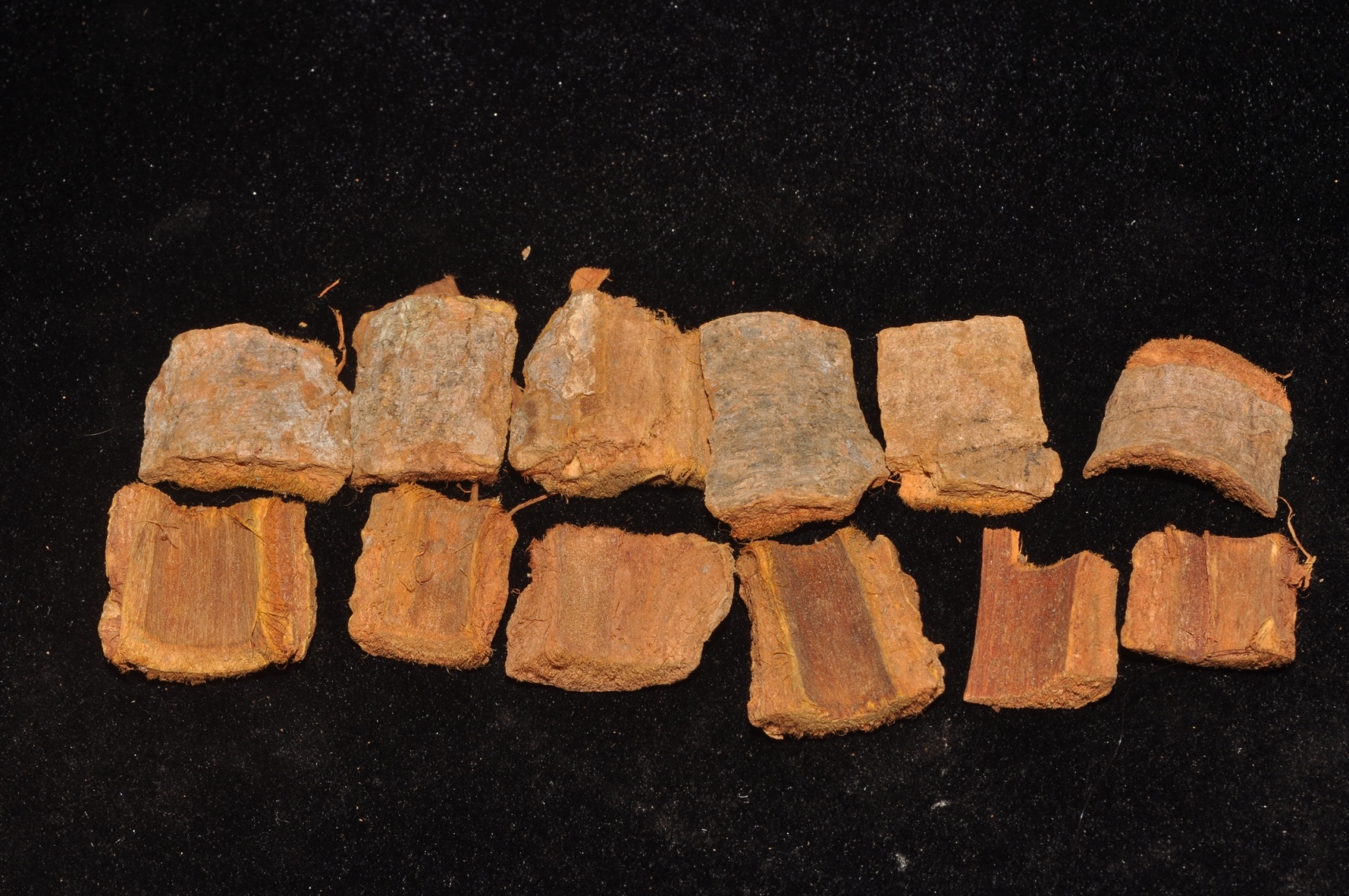

Supplement: Supplementary file 3 [file Data_Sheet_3.zip › Fotor Batch/JPZ44.jpg]

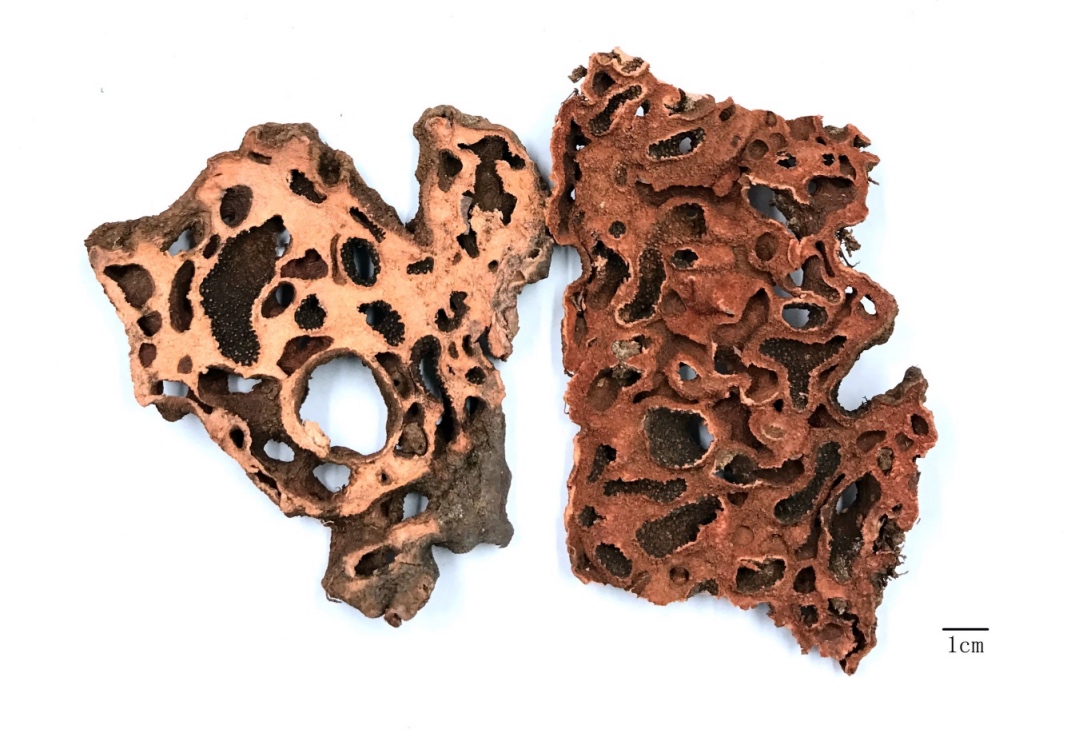

Supplement: Supplementary file 3 [file Data_Sheet_3.zip › Fotor Batch/JPZ50.jpg]
